# A Novel Cell‐Penetrating Lipopeptide for DNA Binding, Self‐Assembly, and Delivery Into HeLa and Pluripotent Stem Cells

**DOI:** 10.1002/psc.70085

**Published:** 2026-01-19

**Authors:** Lucas R. de Mello, Tâmisa Seeko Bandeira Honda, Valeria Castelletto, Patrícia Terra Alves, Sang Won Han, Emerson Rodrigo da Silva, Ian W. Hamley

**Affiliations:** ^1^ School of Chemistry, Food Biosciences and Pharmacy University of Reading Reading UK; ^2^ Departamento de Biophysics Federal University of São Paulo‐ Vila Clementino São Paulo São Paulo Brazil; ^3^ Department of Immunology Institute of Biomedical Science, University of São Paulo São Paulo São Paulo Brazil; ^4^ Division of Nephrology, Department of Medicine Federal University of São Paulo São Paulo Brazil

## Abstract

A lipopeptide is designed that contains an epitope from simian virus T‐antigen (SV40T, PKKKRKV) conjugated to an N‐terminal palmitoyl (C_16_‐) moiety, with the aim to act as an effective cell‐penetrating lipopeptide, with additional aggregation propensity conferred by the lipid chain. A combination of cryo‐TEM and small‐angle X‐ray scattering (SAXS) is used to show that the lipopeptide forms micelles, but mixtures with DNA lead to formation of fractal cluster‐like co‐assemblies due to intercalation of the DNA and peptide. Spectroscopic studies using fluorescence and circular dichroism (along with fiber X‐ray diffraction) show that the peptide interacts with DNA and inserts into the groove. Confocal microscopy along with flow cytometry confirms delivery of DNA into both HeLa and mouse embryonic stem cells (mESCs) in pluripotent state, and the system shows excellent cytocompatibility as confirmed by MTT assays. Our data indicate that the lipopeptide may outperform the DNA transfection agent lipofectamine in DNA delivery into these stem cells and it enables DNA delivery into the cytoplasm and nucleus.

## Graphical abstract

A lipopeptide is designed that contains an epitope from simian virus T‐antigen (SV40T, PKKKRKV) conjugated to an N‐terminal palmitoyl (C_16_‐) moiety, which acts as a cell‐penetrating lipopeptide, with additional aggregation propensity conferred by the lipid chain. The lipopeptide enables DNA delivery into the cytoplasm and nucleus.

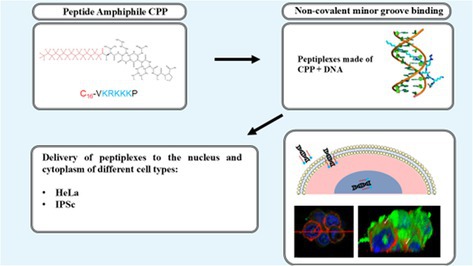

## 1 Introduction

Every year, researchers and the pharmaceutical industry develop new bioactive molecules for a variety of purposes: novel treatments for diseases that challenge existing therapies, vaccines against pathogens of interest, and innovative scaffolds for tissue engineering [1, 2, 3, 4]. However, to exert the desired effect, a molecule first needs to reach its receptor or target, and many biomolecules cannot be internalized by living cells due to factors such as polarity, size, charge, or the lack of a pathway to direct them into intracellular compartments [5]. One key issue in modern therapies is how to bypass the biological membranes of the host and enable the delivery of biomolecules where they are required [6, 7]. The impact of a reliable method of delivery is particularly meaningful for new therapies such as the delivery of nucleic acids, which have been in the spotlight due to DNA and RNA vaccines against COVID, and many other genomic vaccines in development against other viruses or for the treatment of diseases and cancer [8, 9, 10]. One major obstacle for this approach is that nucleic acids are naturally repelled by the cell membrane due to their overall negative charge, consequently resulting in the nucleic acids being processed by endogenous enzymes due to the natural defenses against exogenous nucleic acids from viruses and bacteria [11, 12, 13].

To address these barriers, carrier molecules can be used to bypass membranes and protect nucleic acid cargo. Among the available strategies, cell‐penetrating peptides (CPPs) offer several advantages: low production cost via solid‐phase peptide synthesis, the capacity to form non‐covalent complexes with cargo in aqueous media, improved safety compared to viral vectors, and possibility of straightforward chemical modification to enhance functionality [14, 15, 16]. While CPPs can be highly effective to enhance internalization of biomolecules even by simple co‐incubation, the delivery of longer nucleic acid strands and their nuclear targeting remain challenging. These limitations appear especially in the context of non‐covalent approaches [17, 18]. The non‐covalent approach may be preferred since covalently linking the peptides to each different cargo is a costly and laborious task due to the synthetic chemistry required and possible changes in the desired bioactivity of those molecules [19, 20, 21]. Due to these limitations, novel approaches to improve the delivery of non‐covalent complexes have been developed during the last couple of decades, including sequences with cell penetrating capacities like the WRAP family or sequences isolated from viral proteins such as TAT‐HIV, a CPP derived from the minimal penetrating fragment isolated from the Trans‐Activator of Transcription protein (TAT) [22, 23]. Another strategy in this context is the linkage of fatty acids to the peptide backbone of well‐established CPPs, such as the sequence Pepfect, which consists of the traditional amphiphilic CPP Transportan, N‐terminally stearoylated [24, 25, 26]. The native Transportan 10 (TP10) peptide is a relatively successful CPP which consists of two domains derived from Galanin and the venom wasp related peptide Mastoporan. Addition of a fatty chain to the peptide backbone leads to a lipopeptide that retains the capacity of Transportan 10 to self‐assemble into nanoparticles around the cargo and deliver different classes of nucleic acids inside living cells with enhanced efficiency. This class of lipopeptide consisting of fatty chain linked to a peptide backbone is also called a peptide amphiphile (PA), also termed lipopeptide herein [25, 27, 28, 29].

**SCHEME 1:**
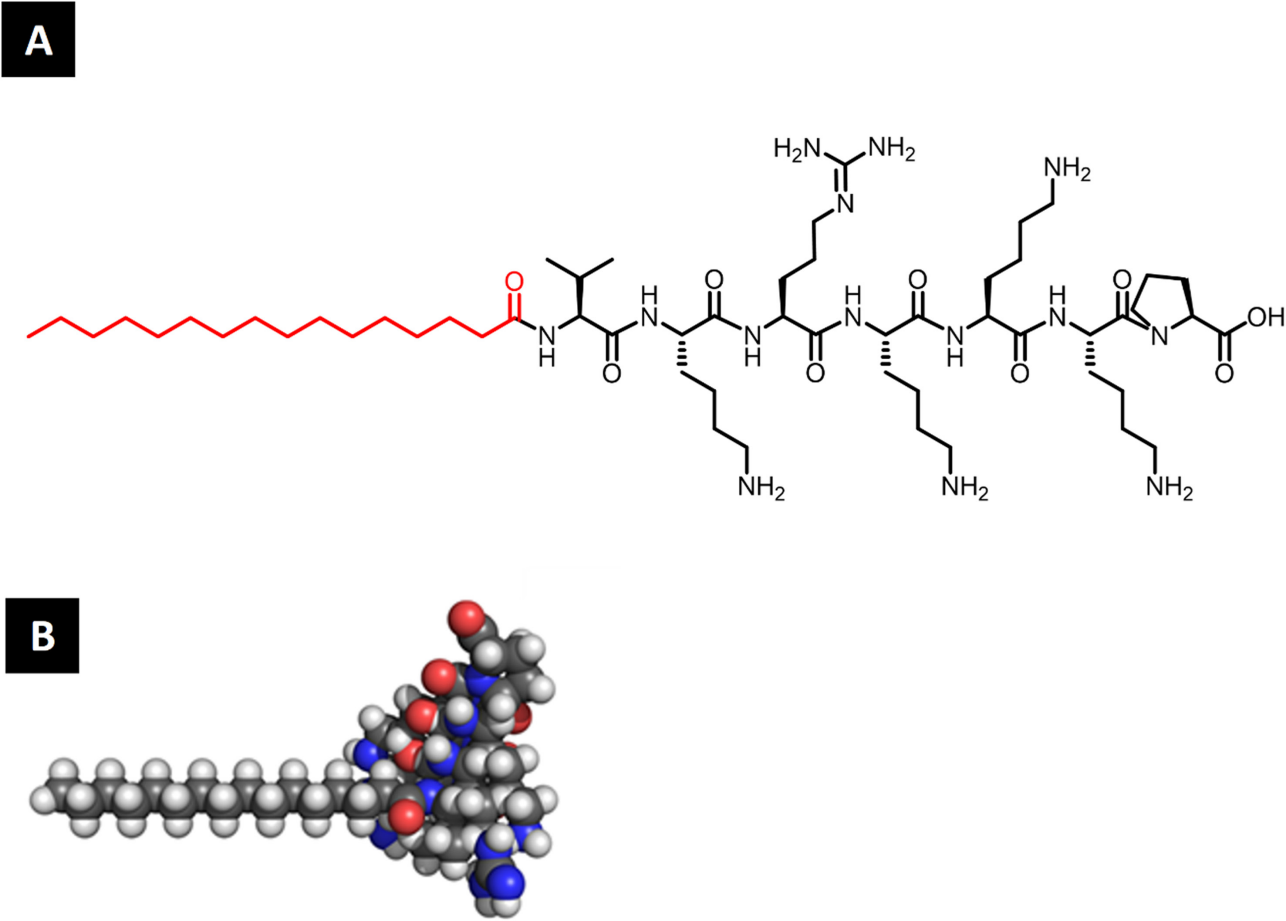
Structure of the peptide amphiphile investigated in this study. (A) Chemical structure of lipopeptide C_16_‐VKRKKKP. The palmitoyl group is highlighted in red, while the peptide backbone is represented in black. (B) Molecular representation of the lipopeptide C_16_‐VKRKKKP.

In this work we introduce a new PA to serve as a CPP, the sequence Palmitoyl‐VKRKKKP, or simply C_16_‐VKRKKKP (Scheme 1). The heptapeptide sequence in this molecule is a nuclear localization sequence derived from the simian virus T‐antigen (SV40T, PKKKRKV), which retains the capacity of internalizing cell membranes and reaching the nucleus [30, 31]. The main objective of this work is to improve the self‐assembly of the original CPP and deliver a nucleic acid cargo into different types of cells, including mouse embryonic stem cells (mESCs) maintained in an artificially naïve state, a cell type of great interest for tissue engineering which can be remarkably challenging as a target for gene delivery [32, 33].

We hypothesized that linking a cationic peptide sequence to a hydrophobic tail would yield an amphiphilic molecule capable of self‐assembling into supramolecular structures, suitable for use as nanocarriers for nucleic acids. To characterize self‐assembly, we employed biophysical techniques including spectrofluorimetric assays and circular dichroism. The morphology and organization of the resulting structures were analyzed using advanced methods such as cryo‐transmission electron microscopy (Cryo‐TEM), atomic force microscopy (AFM), and small‐angle X‐ray scattering (SAXS), allowing us to assess their formation and stability in solution. The biological performance of the lipopeptide was evaluated through cytotoxicity assays, including mitochondrial activity measurements via MTT and flow cytometry. Flow cytometry was also used to quantify the proportion of cells successfully internalized with peptiplexes (non‐covalent complexes between the amphiphile and fluorescently labeled DNA). Confocal microscopy was then applied to visualize the intracellular distribution of these complexes in HeLa cells. Finally, we tested the uptake of peptiplexes by ES‐E14TG2a cells maintained in a pluripotent state, to explore their potential for gene delivery in cell and tissue engineering applications.

## 2 Materials and Methods

### 2.1 Reagents

The PA C_16_‐VKRKKKP (expected *Mw* = 1121.6 g mol^−1^, observed 1123.2 g mol^−1^) was purchased with a purity of 99% by HPLC (shown in Figure S1a) from Peptide Protein Research Ltd. (Fareham, UK). HeLa and ES‐E14TG2a cells were stock cells or purchased from ATCC (Virginia, USA), respectively. Gelatin, 2‐mercaptoethanol, PD 0325901, CHIR99021, and 3‐(4,5‐dimethylthiazol‐2‐yl)‐2,5‐diphenyltetrazolium bromide (MTT) were purchased from Sigma Aldrich (St. Louis, USA). For the assays containing fragmented dsDNA, calf thymus DNA (Sigma‐Aldrich, USA) was fragmented using a Diagenode Bioruptor 300 (Liège, Belgium). Solutions containing 6 mg·mL^−1^ were loaded in 300 μL microtubes and submitted to ultrasonication in the Bioruptor chamber, filled with a water+glass mixture, using 30/60 s on/off cycles. The vials were rotated at about 5 rpm to homogenize temperature, and the total sonication time was about 4 h to obtain fragments in the 100‐ to 200‐bp range, as attested by agarose electrophoresis. The DNA fragments were labeled with YOYO‐1 by incubating the nucleic acids with the dye at a 4700:1 base‐pair–to–dye ratio in the dark, at room temperature, for 1 h. The large excess of base pairs relative to dye was used to minimize the presence of unbound YOYO‐1 in the downstream assays. After incubation, the labeled material was lyophilized to ensure extended shelf life. The molecular weight of DNA was taken to be 660 g·mol^−1^, corresponding to the average mass of one base pair.

### 2.2 Atomic Force Microscopy (AFM)

The samples for AFM imaging were prepared by casting small droplets of PA or PA/DNA solutions prepared under the same conditions as the cellular assays onto freshly cleaved mica surfaces, which were then left to dry overnight inside desiccators. Imaging was performed using a Bruker Multimode 8 microscope at the National Nanotechnology Laboratory (LNNano, Campinas, Brazil). The instrument was operated in tapping mode and used to scan surface areas of 1 × 1 μm^2^, producing topographical images with a resolution of 512 × 512 pixels. The set‐up was enclosed inside a chamber under a constant nitrogen stream to keep humidity below 5% at room temperature 20°C. Image treatment was performed with the software Gwyddion.

### 2.3 Circular Dichroism

CD spectra were obtained using a Chirascan spectropolarimeter (Applied Photophysics, UK). For the titration, a quartz cuvette of 1‐mm pathlength was first filled with a solution containing only the PA and the titration was done by adding small volumes of a highly concentrated DNA solution. We also performed titrations consisting of cuvettes containing DNA with the addition of the PA to better understand the induced circular dichroism caused in the DNA structure by the ligand. The resulting spectra were processed by using the Chirascan built‐in software, ProData Viewer, and Origin.

### 2.4 Cell Culture and Fluorescence Imaging by Confocal Microscopy

HeLa cells were cultured in Dulbecco's modified Eagle medium (DMEM) with 10% fetal bovine serum and 2 mM of glutamine (ThermoFisher Scientific, Massachusetts) and maintained in a controlled atmosphere with 5% CO_2_ at 37°C. For the assays with stem cells, ES‐E14TG2a mouse embryonic stem cells were maintained in DMEM with the addition of 0.1 mM of 2‐mercaptoethanol as a reducing agent, the MEK/ERK pathway inhibitor PD 0325901 (Sigma‐Aldrich, Missouri), and GSK3 selective inhibitor CHIR99021 (Sigma‐Aldrich, Missouri), both used to inhibit differentiation pathway and maintain pluripotency [28]. The cells were seeded in 24‐well plates coated with 0.1% gelatin and maintained in a controlled atmosphere with 5% CO_2_ at 37°C. In each well, glass coverslips were placed on the bottom, and 5 × 10^4^ cells were added and incubated for 24 h. For the internalization assays, cultures were washed three times with PBS to remove residual serum and cell debris. Peptide stock solutions were prepared at 1 mM in ultrapure water. Complexes were prepared by mixing 5 μg of DNA with the appropriate amount of PA stock to obtain the desired molar ratios in 50 μL of ultrapure water. These mixtures were incubated at 37°C for 30 min and then mixed with 50 μL of DMEM before being added to 900 μL DMEM in a 24‐well plate. A positive control consisting of DNA + Lipofectamine 2000 was prepared and incubated for 4 h at 37°C for internalization. A negative control consisting of DNA alone, at the same concentration, was prepared and incubated under the same conditions. After incubation, samples were washed 3 times with PBS to remove unattached cells and unbonded complexes. Fixation was performed with 4% paraformaldehyde and nuclear staining was carried out with DAPI (Invitrogen, California) in PBS for 5 min at room temperature. Actin filaments were stained with Phalloidin conjugated with Texas‐red (Invitrogen, California). For this, fixed cells were washed three times with PBS, incubated with Triton X‐100 for 15 min at room temperature, washed again, and then incubated with 6 μM of phalloidin Texas‐red at room temperature for 1 h. After staining, cells were washed three times with PBS and mounted on glass slides with anti‐quenching mounting media Fluoromount‐G (ThermoFisher Scientific, Massachusetts). Imaging was carried out using a confocal microscope (Leica TCS SP8, Mannheim, Germany) equipped with laser sources appropriate for each fluorophore's excitation wavelength. Image processing was performed using ImageJ software.

### 2.5 Flow Cytometry

The internalization rate was assessed through flow cytometry. Aliquots of 5 × 10^4^ HeLa cells were cultivated in a 24‐well plate overnight, followed by incubation with peptiplexes for 4 h. Cells were then incubated with Fixable Viability Dye eFLuor 780 from eBioscience (California, USA) according to the manufacturer's instruction. Flow cytometry was performed using a BD LSRFortessa instrument (Model 4.2.4.2 from BD Biosciences, New Jersey). Data analysis was carried out using FlowJo software (version 9.2), and the gate strategy is detailed in Figure S5.

### 2.6 Fluorescence

Fluorescence spectra were recorded with a Cary Eclipse Fluorescence Spectrometer (Agilent Technologies, UK). For this assay, samples with different concentrations of PA were prepared by serial dilution in 50‐μM pyrene (Sigma Aldrich, California). The spectra were processed firstly with the Cary Eclipse software and then analyzed in Origin to determine the CAC concentration.

### 2.7 In Silico Models for Docking

Based on the data acquired, we proposed simple models for the docking of C_16_‐VKRKKKP into a double strand of DNA with random sequence. For this, both sequences were generated in Pymol, and the energy was minimized with at least 1000 steps. After this, both molecules were uploaded to the online server Patchdock. The resulting models were further refined by Firedock software for fast docking interaction refinement, and the best results were confronted with the biophysical data acquired for the interaction between PA/DNA [34, 35, 36, 37, 38].

### 2.8 MTT Assay

Cytotoxicity was assessed using the 3‐(4,5‐dimethylthiazol‐2‐yl)‐2,5‐diphenyltetrazolium bromide (MTT) assay. DMEM, fetal bovine serum, glutamine, and penicillin–streptomycin were purchased from Thermo Fisher Scientific (Massachusetts, USA). 3‐(4,5‐dimethylthiazol‐2‐yl)‐2,5‐diphenyltetrazolium bromide (MTT) was purchased from Sigma‐Aldrich (St. Louis, USA) and dimethyl sulfoxide (DMSO) from LGC Biotecnologia (Sao Paulo, Brazil). HeLa cells (4 × 10^3^ cells, 100 μL per well) were seeded into 96‐well plates and incubated for 24 h with PA and PA/DNA in DMEM supplemented with 10% FBS and 1% glutamine. Cells were then incubated for 4 h with 0.5‐mg/mL MTT. The resulting formazan crystals were dissolved in 100 μL of DMSO under gentle agitation for 45 min at 37°C, protected from light. Absorbance was measured at 570 nm using a SpectraMax J2 microplate spectrophotometer (Molecular Devices, San Jose, USA). Data was plotted and analyzed using GraphPad Prism 8 (GraphPad software, USA).

### 2.9 Small Angle X‐Ray Scattering Assays

The SAXS studies were performed on two beamlines. The first was beamline B21 (Diamond Light Source Ltd., UK) [39], and the second was SWING (SOLEIL, France) [40]. On beamline B21, the samples were placed into the 96‐well plate of an EMBL BioSAXS robot and then injected via an automated sample exchanger into a quartz capillary with dimensions of 1.8‐mm internal diameter positioned in front of the X‐ray beam. The quartz capillary was enclosed in a vacuum chamber during the entire experiment. The flow of the sample through the capillary was continuous during the SAXS data acquisition. Beamline B21 operated with a fixed camera length (3.9 m) and a fixed wavelength (*λ* = 1.00 Å). The images were captured using a Pilatus 2 M detector. The software Scatter was used for the data processing (background subtraction, radial averaging). On SWING, samples were also delivered in a quartz capillary under vacuum in the X‐ray beam using a BioSAXS setup. Data were collected using an in‐vacuum EigerX‐4M detector, with an X‐ray wavelength 1.033 Å at two sample‐to‐detector distances, 6.217 and 0.517 m. Data were reduced to one‐dimensional form as a function of wavenumber *q* = 4*π*sin*θ*/*λ* (where 2*θ* is the scattering angle), averaged and the background subtracted using the software Foxtrot. The data fitting was performed with the help of the free software Sasfit [41, 42, 43].

## 3 Results and Discussion

### 3.1 Defining the Mechanisms for Self‐Assembly by Fluorescence and Circular Dichroism

The first step was to determine if this new molecule would self‐assemble in liquid media, in contrast to the unlipidated peptide PKKKRKV. The self‐assembly of C_16_‐VKRKKKP was assessed using steady‐state fluorescence assays with different concentrations of the PA C_16_‐VKRKKKP in the presence of pyrene, an aromatic probe widely used to study macromolecular aggregation due to its fluorescence sensitivity to microenvironment polarity and the increased quantum yield upon incorporation into aggregates [44, 45, 46, 47]. The critical aggregation concentration (CAC) of C_16_‐VKRKKKP was found to be 0.73 mM (i.e., 0.8 mg/mL) from the concentration dependence of the intensity ratio of vibronic peaks I_373_/I_383_ of pyrene (Figure S2).

**FIGURE 1:**
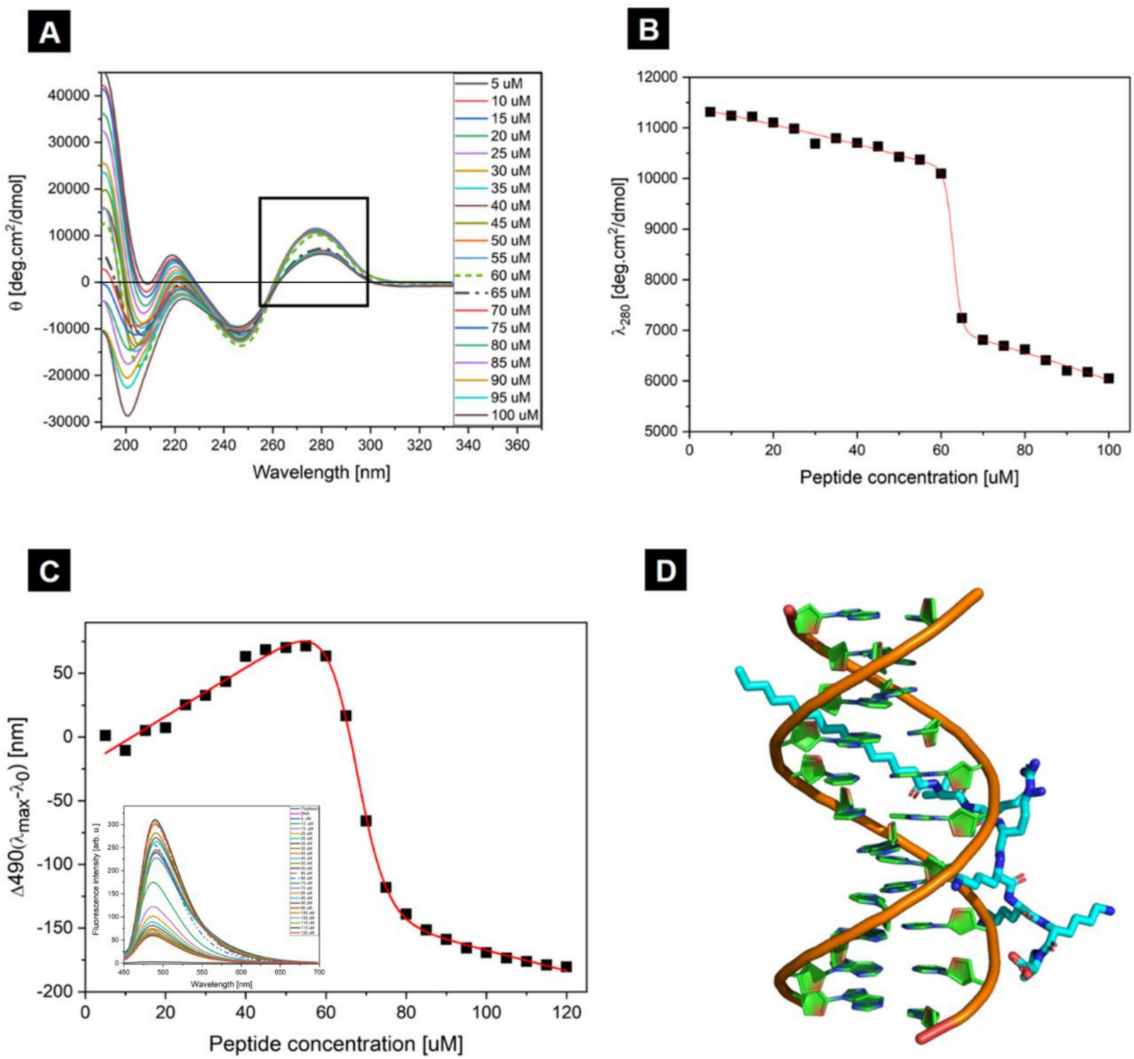
(A) CD spectra of titrations made by adding small volumes of a highly concentrated PA stock in a cuvette initially containing 100‐μM DNA. The curves corresponding to 60‐ and 65‐μM PA are depicted differently as dashes and dash‐dotted lines, respectively. (B) Plot of the ellipticity at 280‐nm fitted with a bi‐dose response function. (C) Thioflavin fluorescence of a titration assay similar to that performed in CD measurements. The plot shows the difference of emission intensity between each titration and the initial DNA solution. The data have been fitted with a bi‐dose response model. (D) Molecular model depicting the non‐covalent interaction between dsDNA and PA.

After determining that this PA self‐assembles in aqueous solution, the next step was to observe the secondary structure and any modifications due to the presence of dsDNA by circular dichroism (Figure 1). Mixing equimolar solutions (1 mM) of peptide and dsDNA produced CD spectra that could not be reproduced by a linear combination of the individual components, indicating direct interaction and assembly into complexes with altered secondary structure (Figure S3). To better explain this effect, we performed a titration starting from a solution of 100 μM of DNA to which a highly concentrated solution of PA was progressively added, resulting in final concentrations of 100 and 50 μM of PA and DNA, respectively. The resulting CD spectra (Figure 1A) can be separated into two regions of interest: below 240 nm there are contributions from the peptide and DNA, and between 240 nm and 340 nm, the signal is primarily from the double‐helix strand of DNA [48, 49]. Initially, the spectra show the classical B‐DNA pattern, with positive bands near 190, 220, and 280 nm. Progressive peptide addition caused a steady decrease in the ellipticity at 280 nm, culminating in a sharp drop for addition of 60‐ to 65‐μM PA. This point coincided with the near molar equivalence between dsDNA and PA (70‐μM DNA, 60‐μM PA) and corresponds to an amine‐to‐phosphate charge ratio (N^+^/P^−^) ~ 2.1. The sigmoidal shape of the curve indicates cooperative binding between PA and DNA, while N^+^/P^−^ ~ 2.1 indicates that stoichiometric excess of positive charges is necessary to achieve full complexation. The spectra at these two points are highlighted as dashed lines in Figure 1A [50, 51].

The transition occurs when there is an excess of C_16_‐VKRKKKP relative to DNA. Plotting molar ellipticity at 280 nm against peptide concentration (Figure 1B) yielded a good fit to a bi‐phasic dose–response model. In this model, the decrease at the 280 nm ellipticity could be attributed to PA molecules binding into the groove of B‐DNA, a phenomenon similar to that observed with groove binding molecules like DAPI, which also induces a reduction in the 280‐nm band [52]. In this case, our data suggest that above a certain concentration there may be interaction between the DNA and the PA, where it preferably binds into the groove region.

To further clarify the aggregation process of the PA and DNA into peptiplexes we performed a titration in the presence of Thioflavin T. Thioflavin T is a probe classically used for the detection of *β*‐amyloid fibers [53, 54]; however, ThT also has the capacity to bind into the groove of B‐DNA enhancing the fluorescence of this dye after intercalation due to a favorable microenvironment, and the formation of ThT duplexes fitting in the groove. In this assay, a cuvette containing 100 μM DNA in a 50 μM ThT aqueous solution was titrated with concentrated C_16_‐VKRKKKP. The fluorescence change, Δ*I*(490 nm) = *I*_max_ − *I*_0_, was calculated for each addition, where *I*_max_ is the fluorescence after peptide addition and *I*_0_ is the initial ThT fluorescence in DNA solution without lipopeptide. As shown in Figure 1C, the fluorescence increased steadily with each peptide addition up to 60 μM C_16_‐VKRKKKP, reaching a maximum at molar equivalence between DNA and PA. A sharp transition occurred at 65 μM, above which fluorescence decreased. This decline is consistent with peptide‐induced displacement of ThT from the DNA groove, as free ThT in solution exhibits a lower quantum yield than DNA‐intercalated ThT [55]. The data were successfully fitted to a bi‐phasic dose–response model. The observed behavior and transition points were consistent across both circular dichroism and ThT fluorescence assays, indicating a non‐covalent interaction between the PA and the groove of dsDNA.

To explore this interaction at the molecular level, we used the PatchDock online docking server with a randomly generated B‐DNA helix (modeled in PyMOL) and the C_16_‐VKRKKKP structure. The resulting *in silico* model suggests that the lipopeptide binds within the DNA groove through electrostatic interactions between the phosphate backbone and the peptide's cationic residues. This type of binding has been reported previously for other cationic peptides [35, 56, 57]. A representative molecular model is shown in Figure 1D.

**FIGURE 2:**
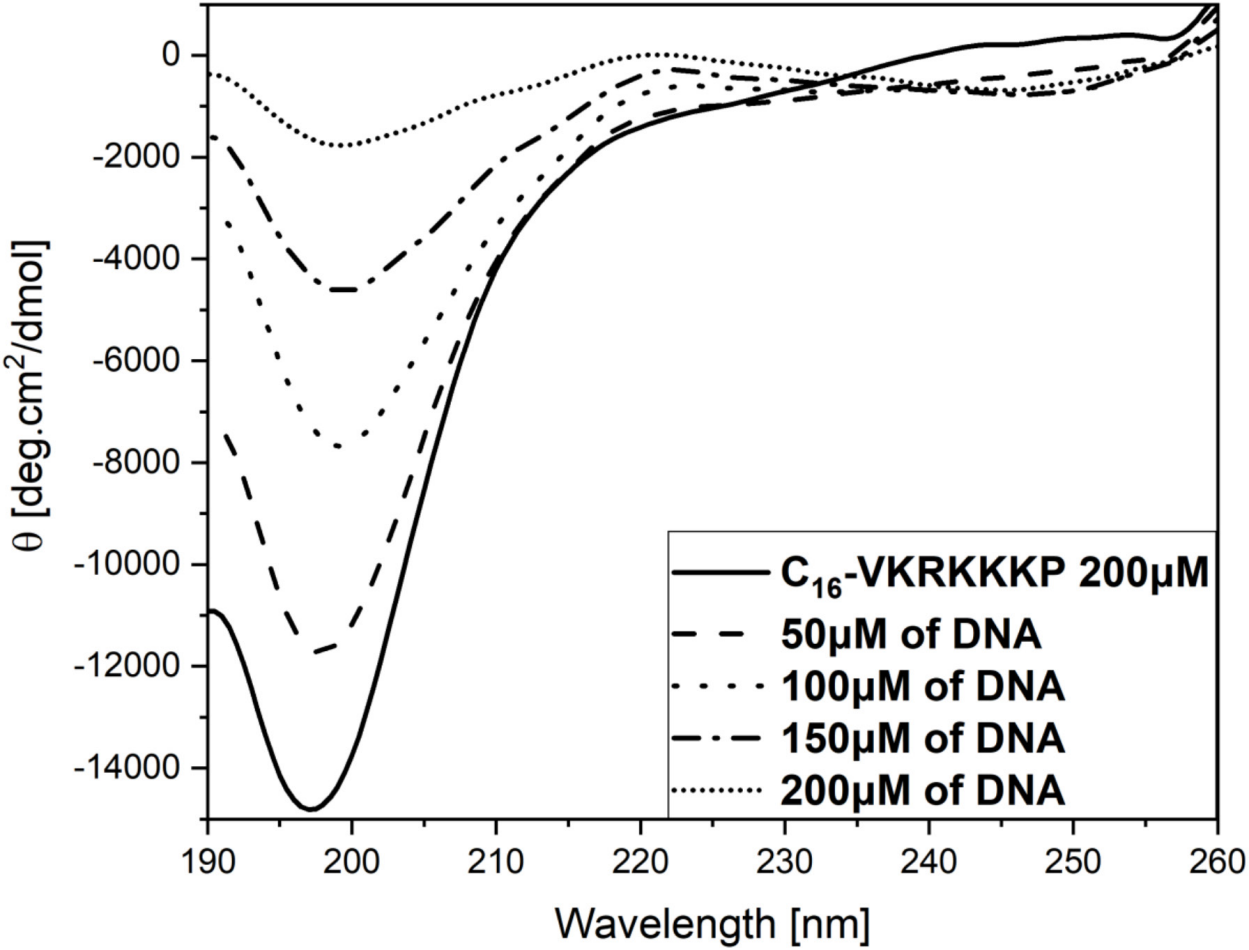
Circular dichroism spectra for 200‐μM solution of PA titrated with a more concentrated solution of DNA until reaching an equivalent molar concentration. The corresponding curves are: 200 μM PA (continuous line); 200 μM PA + 50 μM DNA (dashed line), PA + 100 μM of DNA (short dashed line), PA + 150 μM DNA(dash and dot line), and PA + 200 μM DNA(only dots).

To better clarify the participation of the PA in forming the peptiplexes and its impact on the secondary structure, we also evaluated modifications in the secondary structure via a second titration monitored by CD. The impact of adding DNA in a cuvette containing a surplus of C_16_‐VKRKKKP was examined, paying special attention to the region below 260 nm. For a solution containing 200 μM of peptide (Figure 2, continuous line), the CD spectrum indicates an unordered secondary structure as evidenced by a strong negative minimum around 190 nm, a classical random coil structure profile [50, 51].

However, increasing the DNA concentration in the sample altered the spectra, suggesting structuration and ordering of the lipopeptide in the presence of DNA. This was evidenced by the suppression of bands near 190 nm and 215–220 nm, both typically associated with unordered secondary structures. Such modifications indicate the structuring of DNA and PAs into peptiplexes, potentially comprising a mixture of unordered regions with a minor *β*‐sheet component [58].

#### 3.1.1 Structural Analysis

**FIGURE 3:**
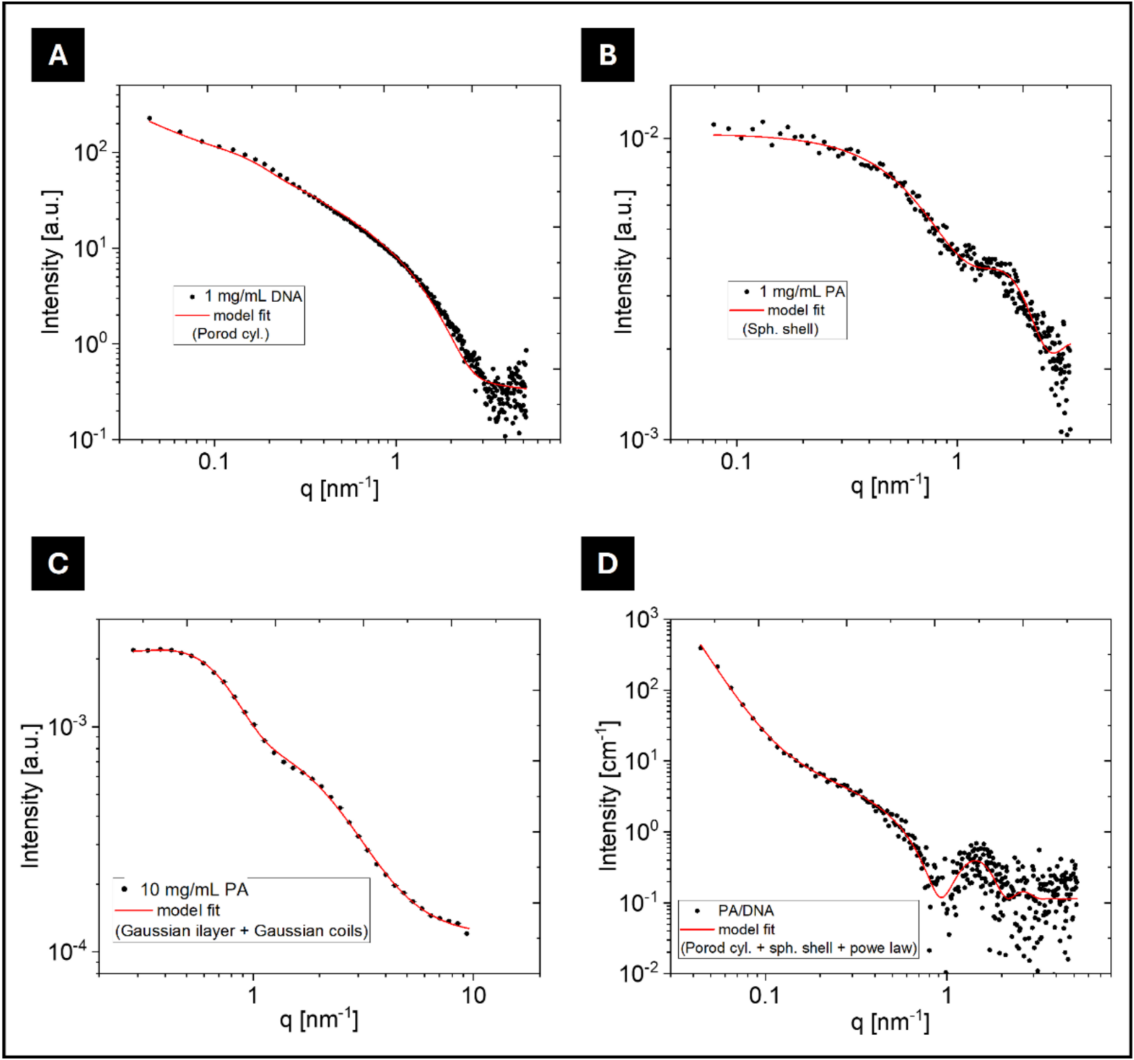
(A–D) Synchrotron SAXS data for (A) DNA, (B) lipopeptide, (C) lipopeptide at higher concentrations, and (D) lipopeptide/DNA complexes, with red curves representing model fits.

To investigate the nanoscopic structure of complexes between the peptide and nucleic acids, SAXS was performed with solutions containing PA, DNA, or a mix of PA/DNA (Figure 3A–D).

SAXS experiments provide unique in situ information on the local structure of our aggregates and offer insights into how the system self‐assembles from small subunits into larger fractal aggregates [59, 60, 61]. The data for DNA were well‐fitted using a Porod cylinder model, yielding a radius (R) of 1.1 ± 0.3 nm, consistent with the expected radius of a DNA chain in water [62, 63].

Consistent with a micellar structure at low concentration, SAXS data for C_16_‐VKRKKKP at 1 mg/mL (Figure 3B) and 10 mg/mL (Figure 3C) were fitted with a spherical shell form factor and a bilayer + Gaussian chain model, respectively. At 1 mg/mL, the PA self‐assembles into spherical shell structures with an outer radius *R*_*o*_ = 2 ± 0.4 nm and a shell thickness of 0.3 nm (Table S1). At 10 mg/mL, SAXS shows that the PA further aggregated into containing a bilayer of lipopeptide. The bilayer head‐to‐head distance is *t* = 5.5 nm (fit parameters in Table S1). A Gaussian coil form factor was included in the fit to allow for unaggregated lipopeptides [64]. The SAXS profile shown in Figure 3D, corresponds to a PA/DNA sample at a 3:1 ratio, and was fitted with a Porod cylinder + spherical shell model. The Porod cylinder form factor has a fixed cylinder length L = 100 nm and a cylinder outer radius *R*_o_ = 3.5 nm.

**FIGURE 4:**
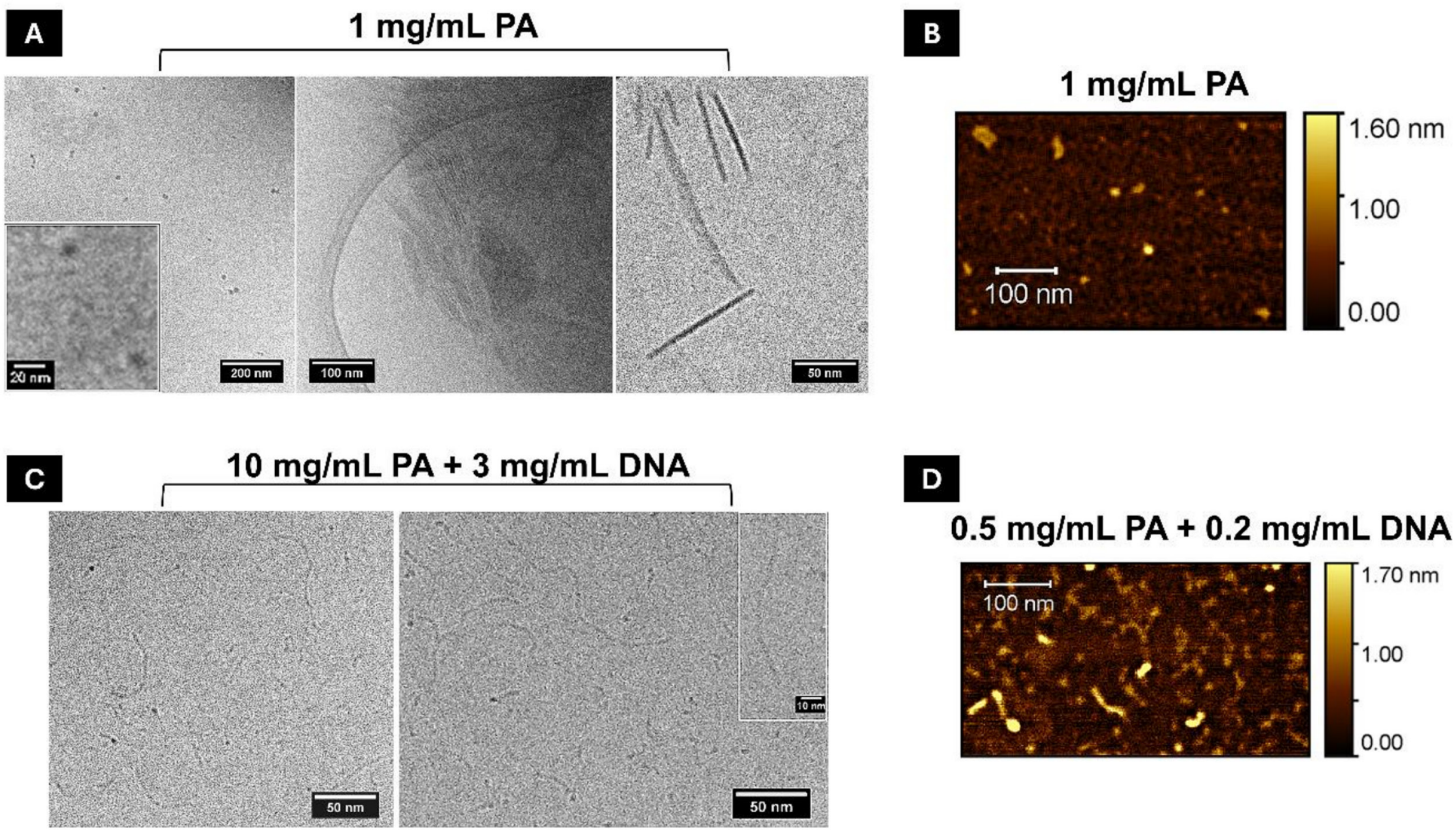
(A) Cryo‐TEM image of a sample comprising C_16_‐VKRKKKP (1 mg/mL), (B) AFM image of C_16_‐VKRKKKP (1 mg/mL) sample, (C) cryo‐TEM image of a sample comprising C_16_‐VKRKKKP + DNA at the concentrations indicated, and (D) AFM image of C_16_‐VKRKKKP + DNA sample at the indicated concentrations.

Cryo‐TEM (Figure 4A) and AFM (Figure 4B) imaging further confirmed the presence of ~10 nm diameter micelles in less concentrated samples along with larger tape‐like extended structures. Samples containing PA/DNA at higher concentrations were also imaged by Cryo‐EM (Figure 4C), revealing both small micelles and longer fibril‐like structures. AFM images at lower concentrations (Figure 4D) show similar micelles, along with shorter worm‐like structures, indicating a possible concentration dependent self‐assembly [65, 66]. We ascribe the micelles to PA assemblies and the fibril‐like structures to PA/DNA co‐assemblies containing extended DNA. Complementing the cryo‐TEM and AFM imaging, PA/DNA solutions were dried between two stalks to produce fibers, enabling fiber XRD measurements to further investigate the nanoscopic arrangement (Figure S4). The resulting 1D scattering profile displayed a prominent peak with a d‐spacing of 4.2 Å. This distance has previously been associated with amphiphile packing in bilayers [67], suggesting a similar packing mode may occur within the peptiplexes.

#### 3.1.2 In Vitro Delivery in HeLa Cells

Spectroscopic and structural studies described above indicated that C_16_‐VKRKKKP successfully formed peptiplexes when co‐solubilized with nucleic acids using a non‐covalent approach. The next step was to evaluate the potential of this new lipopeptide as a CPP. Previous studies have reported improved CPP internalization via the non‐covalent approach when complexes are prepared with an excess of DNA to lipopeptide. To assess this, we investigated the capacity of C_16_‐VKRKKKP to deliver YOYO‐1 labeled DNA fragments (around 200 bp) into HeLa cells, following a protocol previously applied in CPP studies [21, 68, 69, 70]. For this assay, HeLa cells were incubated with 7.6 μM (5 μg) labeled DNA and PA C_16_‐VKRKKKP + DNA at a 2:1 peptide/DNA molar ratio. Control groups included cells incubated with 7.6 μM labeled DNA, the unmodified SV40 peptide + DNA at a ratio of 2:1 as a non‐lipidated CPP control and lipofectamine 2000 + 7.6 μM labeled DNA to compare the internalization of our molecule with a commercial reagent.

**FIGURE 5:**
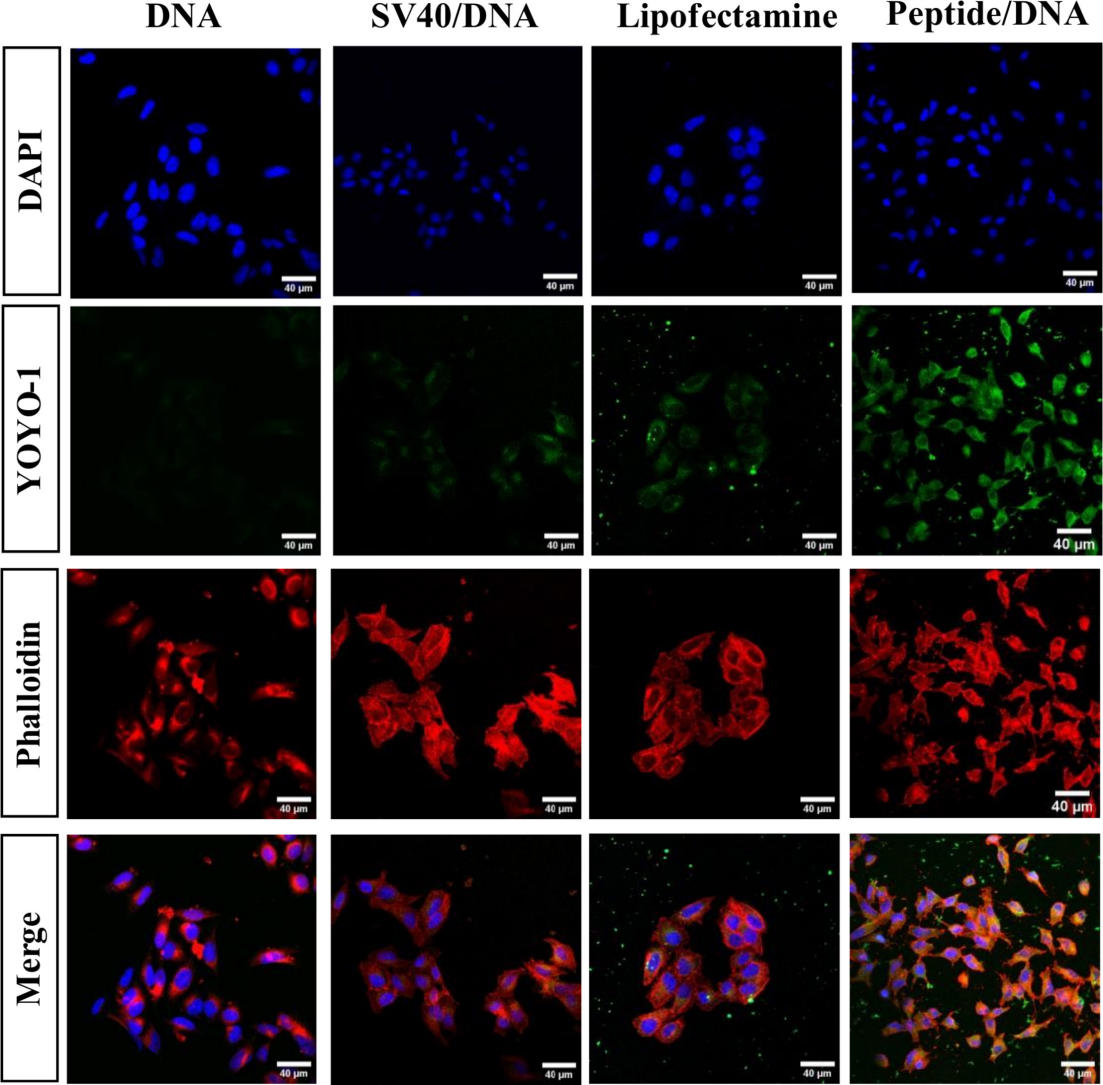
Fluorescence confocal microscopy of HeLa cells incubated with C_16_‐VKRKKKP/DNA complexes (2:1 M ratio) and controls. Channels for individual fluorophores are indicated in the micrographs: DAPI stains nuclei, phalloidin labels actin filaments in the cytoskeleton and YOYO‐1 labels the DNA reporter. The last row shows the merged channels for comprehensive visualization of the cells. Each column represents images obtained after 4 h incubation with DNA only, the native SV40 (PKKKRKV) sequence + DNA, Lipofectamine 2000/DNA, and the last column is C_16_‐VKRKKKP/DNA.

Figure 5 shows no detectable internalization of the fragmented DNA in either the cytoplasm (stained with phalloidin‐Texas red) or the nuclei (marked with DAPI) of HeLa cells incubated only with YOYO‐1 labeled DNA in the absence of carriers. Cells incubated with the unmodified SV40 (sequence PKKKRKV) did not present significant increase in brightness for the green channel, indicating that degree of internalization is poor in this case. In contrast, cells incubated with Lipofectamine 2000/DNA or C_16_‐VKRKKKP displayed a clear intracellular localization of the labeled DNA.

**FIGURE 6:**
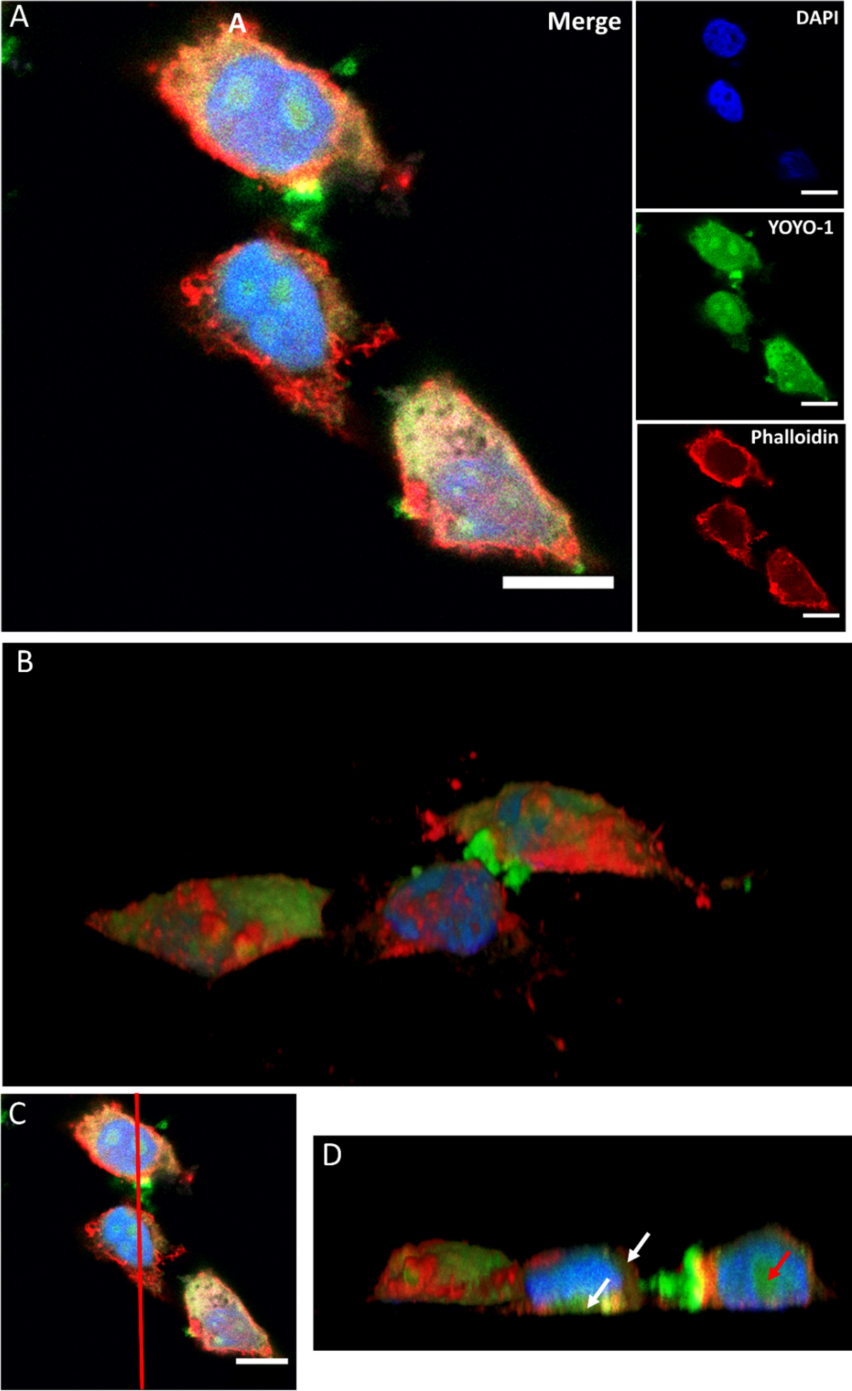
Confocal microscopy of HeLa cells incubated with C_16_‐VKRKKKP/DNA complexes for 4 h. (A) Merge image and 4 separate channels showing fluorescence from dyes labeling different cell structures. (B) 3D reconstruction of the merged image. (C,D) Cross‐section (indicated by red line) of the 3D reconstruction, showing “pockets” of labeled DNA within the cytoplasm and nuclei. White arrows indicate DNA in the cytoplasm, and the red arrow indicates DNA within the nuclei. Scale bar: 10 μm.

Confocal microscopy allows the collection of multiple focal planes to reconstruct a 3D model of the sample. In the case of HeLa cells, this approach enabled high‐resolution imaging of intracellular compartments at a micrometric scale (Figure 6). Figure 6A shows HeLa cells incubated with C_16_‐VKRKKKP/DNA at 400X magnification and Figure 6B shows the three‐dimensional reconstruction of this image. A cross‐section (indicated in Figure 6C) revealed distinct “pockets” of labeled DNA within both the cytoplasm (Figure 6D, white arrows) and the nuclei (Figure 6D, red arrows). This accumulation of peptiplexes in the nucleus, particularly within the nucleolus, has been observed in previous works involving cationic CPPs [71, 72]. It is important to note that YOYO‐1 is a membrane‐impermeable dye and its fluorescence requires intercalation into double‐stranded DNA. Therefore, the fluorescence observed in the cytosol indicates the presence of exogenous DNA fragments inside the cells. The labeled DNA alone (negative control) produced no intracellular signal, confirming that cellular entry requires a carrier. In addition, YOYO‐1 is cationic, making direct association with our positively charged lipopeptide unlikely. The nuclear pattern observed with C_16_‐PKKKRKV was also not detected with Lipofectamine control, suggesting that the lipopeptide modulates the permeability of the eukaryotic barrier to allow the passage of the exogenous DNA or, at least, the migration of the otherwise impermeable dye.

#### 3.1.3 Flow Cytometry and MTT Assays

Following the successful demonstration of peptiplex delivery into living cells by C_16_‐VKRKKKP, the next step was to quantify the percentage of YOYO‐1‐positive cells. Flow cytometry assays were conducted under the same conditions as the fluorescence microscopy assays by incubating the samples with 5 μg of fragmented and YOYO‐1 labeled DNA in four different conditions: DNA alone, DNA complexed with Lipofectamine 2000, and DNA complexed with either the original non‐lipidated SV40 (PKKKRKV) peptide or the lipopeptide C_16_‐VKRKKKP. After incubation, cells were stained with Fixable Viability Dye eFluor 780 to assess cell viability, fixed with PFA, and scanned at the corresponding excitation/emission wavelengths for YOYO‐1 and eFluor 780 to evaluate both DNA internalization and cell viability.

**FIGURE 7:**
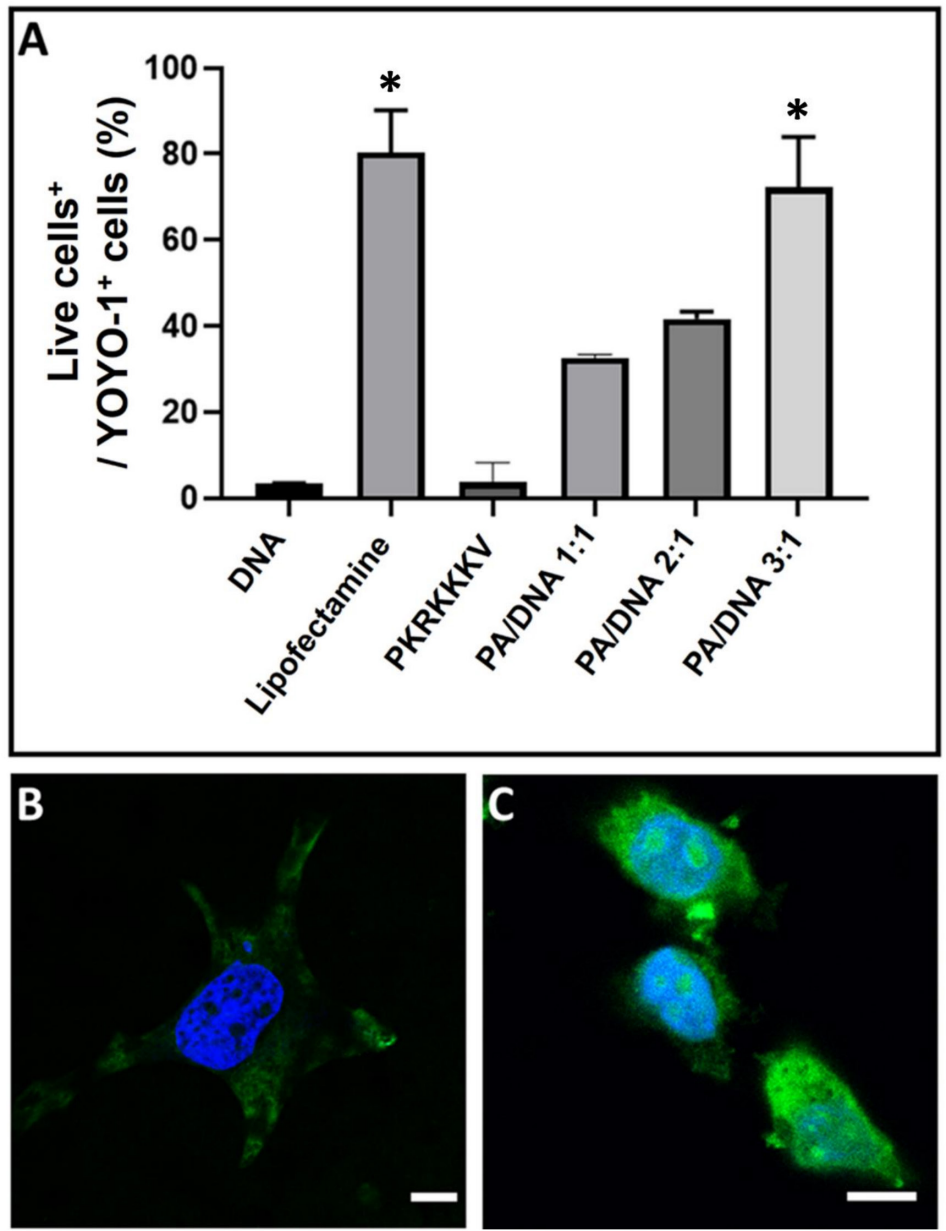
(A) Flow cytometry chart of viable HeLa cells positive for the delivery of YOYO‐1 labeled DNA fragments. Confocal microscopy images: (B) Translocation of YOYO‐1 labeled DNA (green) into the cytoplasm and nuclei (blue, DAPI‐ stained) using the original SV40 peptide and (C) HeLa cells incubated with C_16−_VKRKKKP/DNA peptiplexes. The scale bar corresponds to 10 μm. * = *p* ≤ 0.05, Kruskall–Wallis analysis, *n* = 3.

As shown in Figure 7, cells treated with DNA alone or SV40/DNA peptiplexes at a 2:1 M ratio displayed negligible YOYO‐1 fluorescence. In contrast, treatment with C_16_‐VKRKKKP/DNA peptiplexes at 1:1 and 2:1 M ratios resulted in markedly higher percentages of YOYO‐1–positive cells, with at least a 30% increase in internalization compared to the parent SV40 peptide after 4 h of incubation. This enhanced delivery was also evident in confocal microscopy: samples incubated with SV40/DNA (Figure 7B, 2:1 ratio) exhibited only residual YOYO‐1 fluorescence, whereas C_16_‐VKRKKKP/DNA (Figure 7C, 2:1 ratio) showed clear intracellular labeling. The gating strategy and criteria used for this analysis are detailed in Figure S5, with sample‐specific gating parameters shown in Figure S6.

Cytotoxicity and cell metabolism were further evaluated by MTT assays after 24 h incubation. Baseline cell viability was established by incubating HeLa cells in DMEM alone for 24 h. The resulting bar plots (Figure S8) indicate ≥ 70% cell viability at concentrations of 20 μg/mL or lower for C_16_‐VKRKKKP/DNA peptiplexes. These findings are consistent with the flow cell cytometry and confocal microscopy data, as cells incubated with 1:1 and 2:1 C_16_‐VKRKKKP/DNA ratios showed fewer morphological changes. Such changes, including alteration in the cytoskeleton and nucleus, variations in cell size, reduced adhesion, and fewer points of contact between cells, are indicative of cell stress, which was observed in cells incubated with peptiplexes containing a higher ratio of PA to DNA (Figure S7) [73, 74]. The MTT data in Figure S8 show high cytocompatibility with C_16_‐VKRKKKP and a slight but significant reduction in viability at the highest concentration of C_16_‐VKRKKKP/DNA peptiplexes tested.

#### 3.1.4 In Vitro Delivery of DNA to Pluripotent Stem Cells

**FIGURE 8:**
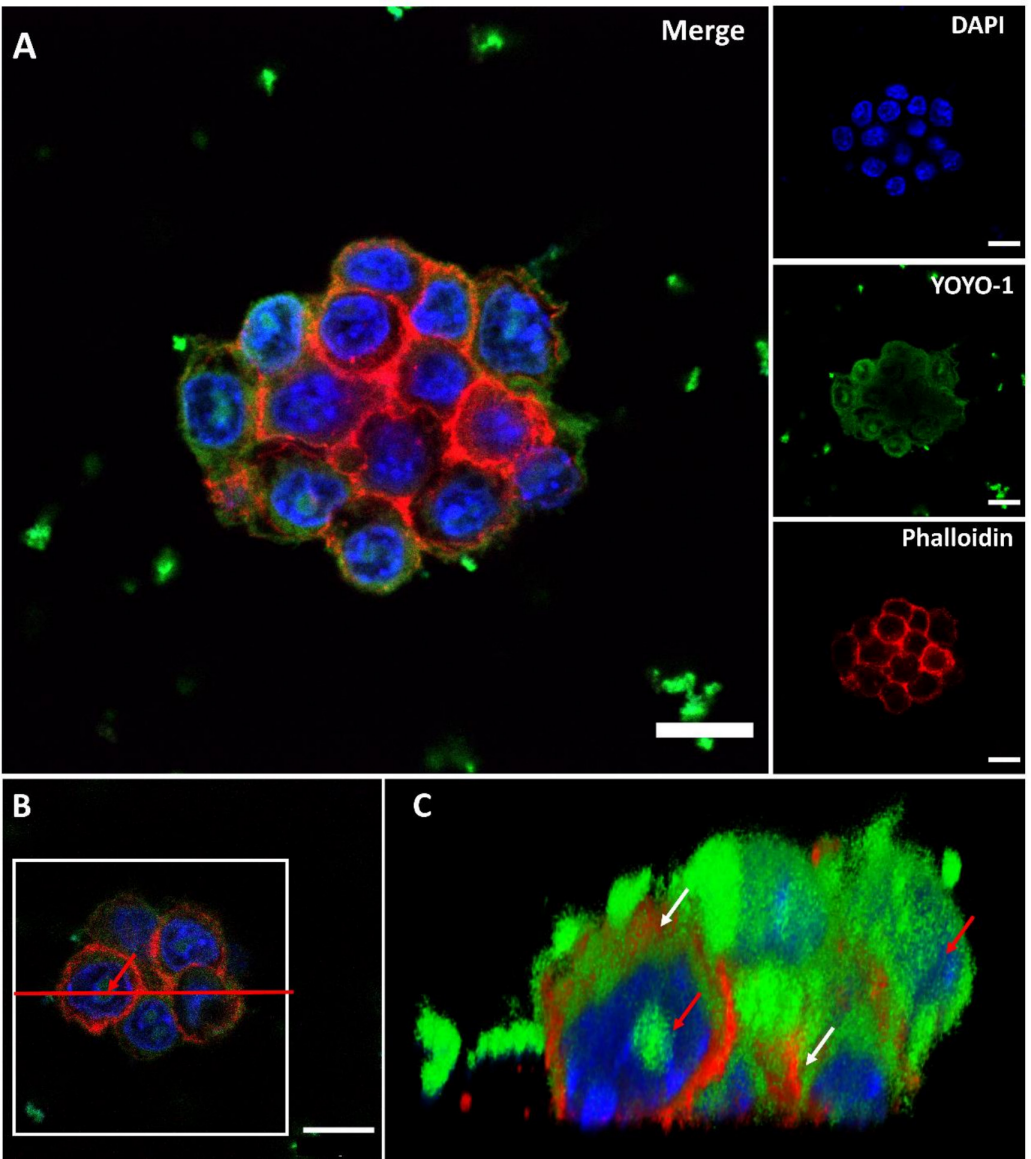
Confocal imaging from ES‐E14TG2a cells incubated with C_16_‐VKRKKKP/DNA complexes for 4 h. (A) Merged image and the 4 channels showing fluorescence from dyes labeling different cell structures. (B,C) Cross‐section of the 3D reconstruction enabling the visualization of “pockets” of labeled DNA delivered into the cytoplasm and nuclei of these cells, similar to the pattern observed in HeLa cells (Figure 6C,D). White arrows indicate pockets of DNA in the cytoplasm and red cells indicates DNA inside the nuclei. Scale bar: 10 μm.

Although the fields of nanomedicine and gene therapy have advanced rapidly with the development of nanocarriers capable of delivering exogenous material into living cells, significant challenges remain for the delivery of nucleic acids into stem cells, including pluripotent stem cells. While these cells can be modified using various viral vectors, the efficiency and stability of transduction are often lower than in many somatic cell types, and maintaining pluripotency during and after transduction can be difficult. Even with optimized non‐viral vectors, efficient cargo delivery to these cells remains more challenging than for differentiated cells [75, 76]. We examined the delivery of YOYO‐1‐labeled DNA into ES‐E14TG2a cells incubated with C_16_‐VKRKKKP under the same conditions used for the internalization assays with HeLa cells. Remarkably, the PA delivered the YOYO‐1 labeled nucleic acids in a pattern of internalization similar to that observed in HeLa cells incubated with C_16_‐VKRKKKP (Figure 8A). In contrast, Lipofectamine 2000 showed poor uptake in ES‐E14TG2a cells when compared with HeLa (Figure S9). A 3D reconstruction obtained by stacking multiple slices along the *Z*‐axis revealed distinct compartments where YOYO1‐labeled DNA was localized (Figure 8B,C). This analysis also demonstrated co‐localization with DAPI in the nucleus and co‐localization with phalloidin that delimited the cytoplasmatic compartment, allowing for precise mapping of DNA distribution within the cells.

A cross‐section of the 3D reconstruction (Figure 8B) shows that C_16_‐VKRKKKP can permeate the cell membrane and deliver the YOYO‐1 labeled DNA not only inside the cytoplasm (Figure 8C, white arrows) but also into the nucleus (Figure 8C, red arrows). When compared with cells incubated with Lipofectamine 2000, it is possible to observe a wider distribution for both the cytoplasm and nuclei (Figure S9). This demonstrates successful delivery of YOYO‐1 labeled DNA into both the cytoplasm and nucleus while preserving the morphology of ES‐E14TG2a [77].

## 4 Conclusions

In this work, we introduced a new PA, C_16_‐VKRKKKP, designed to function as a CPP. We performed a comprehensive biophysical characterization of self‐assembly and delivery capacity in living cells. Unlike its parent peptide, the lipopeptide C_16_‐VKRKKKP self‐assembled into small micelles with nanometer‐scale size distribution in aqueous media, as confirmed by cryo‐EM, AFM, and SAXS. Self‐assembly was also observed in mixtures with DNA, leading to the formation of non‐covalent nanocarriers, named peptiplexes, containing a fragmented model DNA, without requiring covalent linkage or further chemical modifications of either component. Dye fluorescence assays, molecular docking, and circular dichroism analyses revealed the conformation and self‐assembly behavior of the PA, as well as its binding pattern to the DNA groove in a concentration‐dependent manner.

Regarding cytocompatibility, C_16_‐VKRKKKP was well tolerated at the concentrations used in DNA internalization assays, as confirmed by MTT and flow cytometry. The PA successfully delivered cargo into both the cytoplasm and nucleus, as shown by YOYO‐1 fluorescence tracking of DNA. While higher peptide concentrations induced morphological changes in HeLa cells, these effects were minimal at the 2:1 PA/DNA molar ratio used in most assays. The PA is also able to serve as a CPP delivering DNA into pluripotent stem cells (in both the nucleus and cytoplasm), whilst preserving the cell morphology. Overall, these results support the further investigation and development of C_16_‐VKRKKKP as a model CPP with strong potential for use as a nanocarrier for nucleic acids, drugs, or other therapeutic agents, particularly in applications involving stem cells and related biomedical applications. We anticipate that future studies will explore the delivery of bioactive cargoes, such as large plasmid expression vectors and small interfering RNAs, to validate the potential of this lipopeptide for functional gene delivery applications.

## Funding

This work was supported by Engineering and Physical Sciences Research Council (EP/V053396/1) and Fundação de Amparo à Pesquisa do Estado de São Paulo (19/20907‐7, 2019/19719‐1, and 2021/10092‐6).

## Conflicts of Interest

The authors declare no conflicts of interest.

## Supporting information


**Figure S1:** (a) HPLC and (b) ESI‐MS data for C_16_‐VKRKKKP.
**Figure S2:**: Fluorescence assays for different concentrations of peptide in a solution of 50 uM of pyrene, the fluorescence intensity ratio being fitted using a derivative function, which places the CAC around 0.08 wt%, equivalent to 0.7 mM (0.8 mg/mL).
**Figure S3:** (A) CD spectra of DNA 1 mM (black curve), C_16_‐VKRKKKP at 1 mM (red curve). To identify a possible structuration between the molecules, a spectrum was obtained from the addition of the CD spectra of DNA and C_16_‐VKRKKKP at 1 mM curves as a simple simulation of a non‐interacting mixture (blue curve) compared to the measured CD spectrum of a solution containing C_16_‐VKRKKKP/DNA at 1 mM (green curve). (B) Images of samples. Tube **1** contains a solution of C_16_‐VKRKKKP/DNA at 1 mM, and tube **2** contains only the peptide amphiphile. It is possible to observe a difference in turbidity between the two samples, possibly due to phase separation and precipitation in the first tube.
**Figure S4:** 1D plot from a fibre X‐ray diffraction pattern obtained from a sample of C_16_‐VKRKKKP/DNA at a ratio of 2:1. The data shows a prominent peak at 4.2 Å. The initial samples used for fiber production were at a concentration of 10 mg/mL of C_16_‐VKRKKKP.
**Figure S5: Gate strategy used to evaluate the transfection rate (YOYO‐1** ^**+**^ **cells)**. The HeLa cells were selected by complexity (SSC‐A) and size (FSC‐A). The doublets and cell aggregates were excluded using the combination of FSC‐H and FSC‐A. The dead cells were identified and excluded using a live/dead dye labelled with APC‐Cy7. Finally, the Yoyo‐1^+^ cell population were identified as Alexa Fluor 488^+^ by the software FlowJo X, which presents a similar λex/λem. to YOYO‐1.
**Figure S6:**: Dot plots of flow cytometry assays delimiting gates for populations of HeLa cells positive for YOYO‐1 fluorescence (first row) and dead cells positive for the fixable viability dye (second row). The cells were incubated only with DNA (A), peptiplexes at a 2:1 ratio of VKRKKKP/DNA (B), 1:1 C_16_‐VKRKKKP/DNA (C) and 2:1 C_16_‐VKRKKKP/DNA (D). We also observed a difference in the translocation of peptiplexes (dyed in green by the fluorophore YOYO‐1 used to label the fragmented DNA, blue staining of nuclei with DAPI) by confocal microscopy, with a lower degree of internalization by the original VKRKKKP and a higher delivery for C_16_‐VKRKKKP.
**Figure S7:** A) Representative dot plots of viability analysis of cells transfected with different ratios of C_16_‐VKRKKKP. In flow cytometry analysis it was observed that at higher ratios of peptide/DNA (3:1, 5:1 and 10:1), cells underwent further morphogenesis and aggregation, which can be seen in B) in which HeLa cells presented a rounder morphology with a decrease in size and a tendency to aggregation. Those events were excluded from the analysis, resulting in a few events (n) inferior to our cut‐off of 5 × 10^3^.
**Figure S8:**: (A) MTT without DNA. (B) MTT with peptiplexes between C_16_‐VKRKKKP and fragmented DNA. The peptiplexes were slightly more toxic when compared with samples incubated without DNA. There was a significant difference in cell viability between the control the data for 80 μg/mL, as indicated by a Kruskal‐Wallis analysis, *n* = 3 and *p* < 0.05.
**Figure S9:** Confocal images from ES‐E14TG2a cells incubated for 4 h with (A) only DMEM without serum (B) 5 μg of fragmented DNA labelled with YOYO‐1 (C) Lipofectamine 2000 + 5 μg of labelled DNA and in (D), peptiplexes of C_16_‐VKRKKKP/DNA at 2:1 ratio. The samples incubated only with DNA did not present any significant fluorescence of YOYO‐1, contrasting with the strong green fluorescence of samples incubated with DNA and lipofectamine or of C_16_‐VKRKKKP.
**Table S1:** Best fit parameters for SAXS data. A flat background was used in all models. Electron densities were arbitrarily set to −1 and 1, respectively, for cores and shells with an overall intensity scaling factor. R = cylinder radius; R_i_ = inner radius of the spherical shell; R_o_ = outer radius of the shell; R_g_ = gyration radius of the chain; ν = Flory exponent; σ_H_ = width (standard deviation) of the lipopeptide polar head; σ_c_ = width of the bilayer core; *t* = head‐to‐head separation in bilayer.

## Data Availability

The data that support the findings of this study are available from the corresponding author upon reasonable request.
