# Supplementary material for: A Novel Cell‐Penetrating Lipopeptide for DNA Binding, Self‐Assembly, and Delivery Into HeLa and Pluripotent Stem Cells
Source: J Pept Sci. 2026 Jan 19;32(3):e70085. doi: 10.1002/psc.70085 (PMC12815588; doi:10.1002/psc.70085)
Supplement: Supplementary file 1 — Figure S1: (a) HPLC and (b) ESI‐MS data for C16‐VKRKKKP. Figure S2:: Fluorescence assays for different concentrations of peptide in a solution of 50 uM of pyrene, the fluorescence intensity ratio being fitted using a derivative function, which places the CAC around 0.08 wt%, equivalent to 0.7 mM (0.8 mg/mL). Figure S3: (A) CD spectra of DNA 1 mM (black curve), C16‐VKRKKKP at 1 mM (red curve). To identify a possible structuration between the molecules, a spectrum was obtained from the addition of the CD spectra of DNA and C16‐VKRKKKP at 1 mM curves as a simple simulation of a non‐interacting mixture (blue curve) compared to the measured CD spectrum of a solution containing C16‐VKRKKKP/DNA at 1 mM (green curve). (B) Images of samples. Tube 1 contains a solution of C16‐VKRKKKP/DNA at 1 mM, and tube 2 contains only the peptide amphiphile. It is possible to observe a difference in turbidity between the two samples, possibly due to phase separation and precipitation in the first tube. Figure S4: 1D plot from a fibre X‐ray diffraction pattern obtained from a sample of C16‐VKRKKKP/DNA at a ratio of 2:1. The data shows a prominent peak at 4.2 Å. The initial samples used for fiber production were at a concentration of 10 mg/mL of C16‐VKRKKKP. Figure S5: Gate strategy used to evaluate the transfection rate (YOYO‐1 + cells). The HeLa cells were selected by complexity (SSC‐A) and size (FSC‐A). The doublets and cell aggregates were excluded using the combination of FSC‐H and FSC‐A. The dead cells were identified and excluded using a live/dead dye labelled with APC‐Cy7. Finally, the Yoyo‐1+ cell population were identified as Alexa Fluor 488+ by the software FlowJo X, which presents a similar λex/λem. to YOYO‐1. Figure S6:: Dot plots of flow cytometry assays delimiting gates for populations of HeLa cells positive for YOYO‐1 fluorescence (first row) and dead cells positive for the fixable viability dye (second row). The cells were incubated only with DNA (A), peptiplexes at a 2:1 ra [file PSC-32-e70085-s001.docx]

**SUPPORTING INFORMATION**

**C16-VKRKKKP: A Novel CPP for DNA Binding, Self-Assembly, and Delivery into HeLa and Pluripotent Stem Cells**

Lucas R de Mello,^1,2^ Tâmisa Seeko Bandeira Honda, ^3,4^ Valeria Castelletto,^1^ Patricia terra Alves^2^, Sang Won Han^2^, Emerson Rodrigo da Silva^2^, Ian W. Hamley,^1,*^

*^1^ School of Chemistry, Food Biosciences and Pharmacy, University of Reading, Whiteknights, Reading RG6 6AD, U.K.*

*^2^ Departamento de Biophysics, Federal University of São Paulo - Vila Clementino, São Paulo - SP, 04021-001, Brazil*

*^3^ Department of Immunology, Institute of Biomedical Science, University of São Paulo, São Paulo - SP, 05508-000, Brazil*

*^4^Division of Nephrology, Department of Medicine, Federal University of São Paulo, São Paulo, Brazil*

- **CHROMATOGRAPHY AND MASS SPECTROSCOPY DATA**
- **FLUORESCENCE**
- **Circular Dichroism**
- **SAXS FITTING PARAMETERS**
- **CYTOMETRY ASSAYS**
- **MTT ASSAYS**
- **CONFOCAL MICROSCOPY FLUORESCENCE ASSAYS**

**(a)**


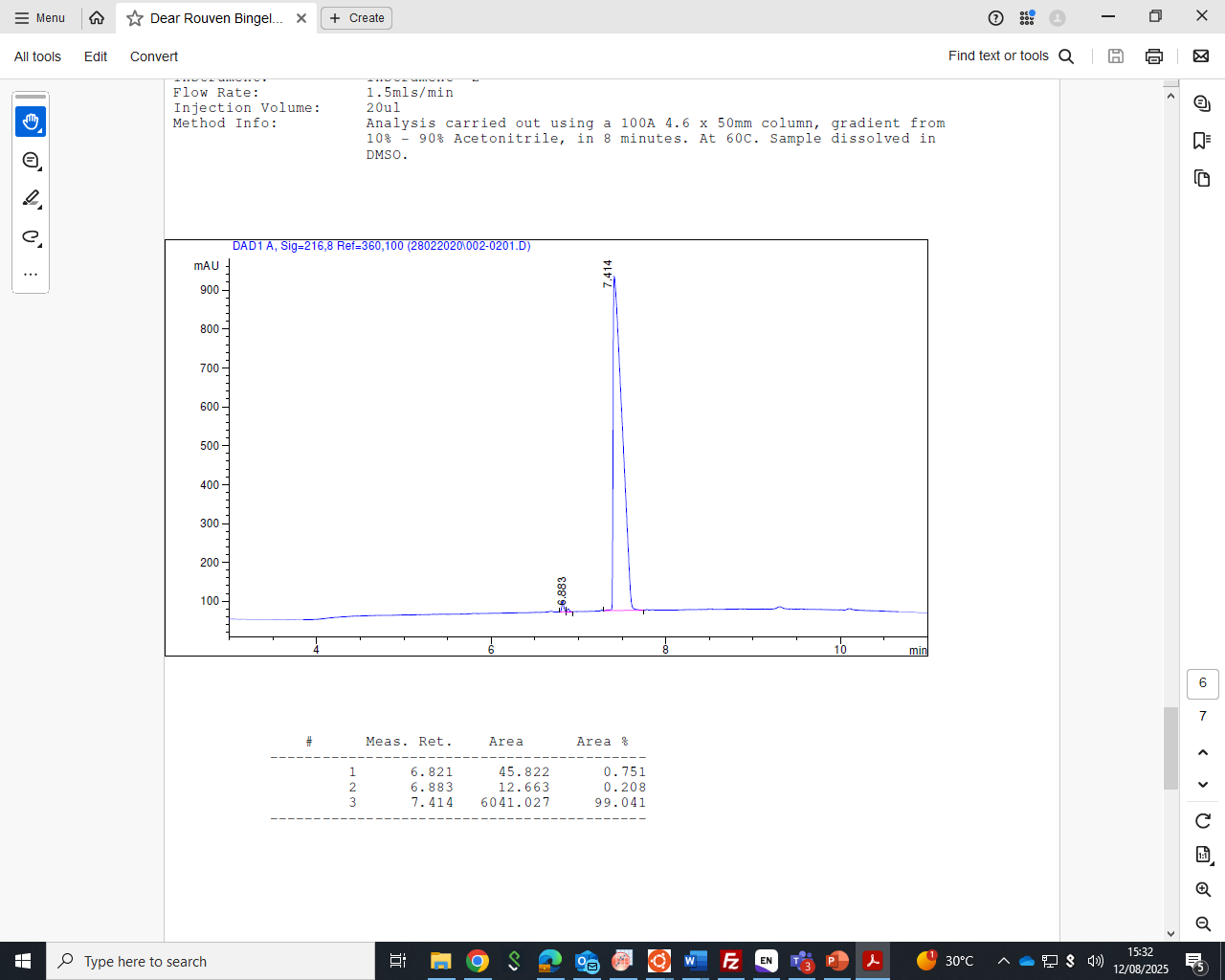


**(b)**


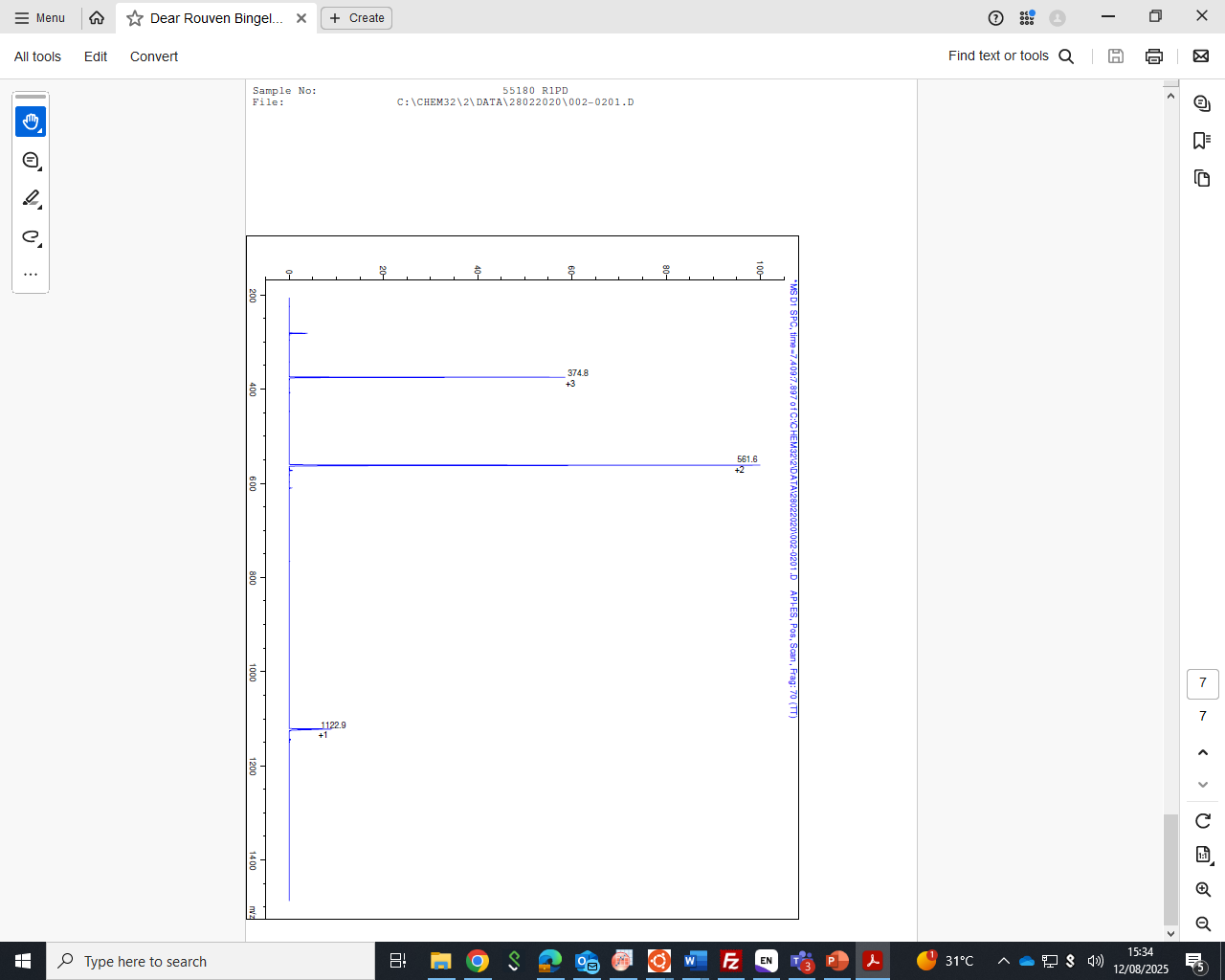


**Figure S1:** (a) HPLC and (b) ESI-MS data for C_16_-VKRKKKP.

**
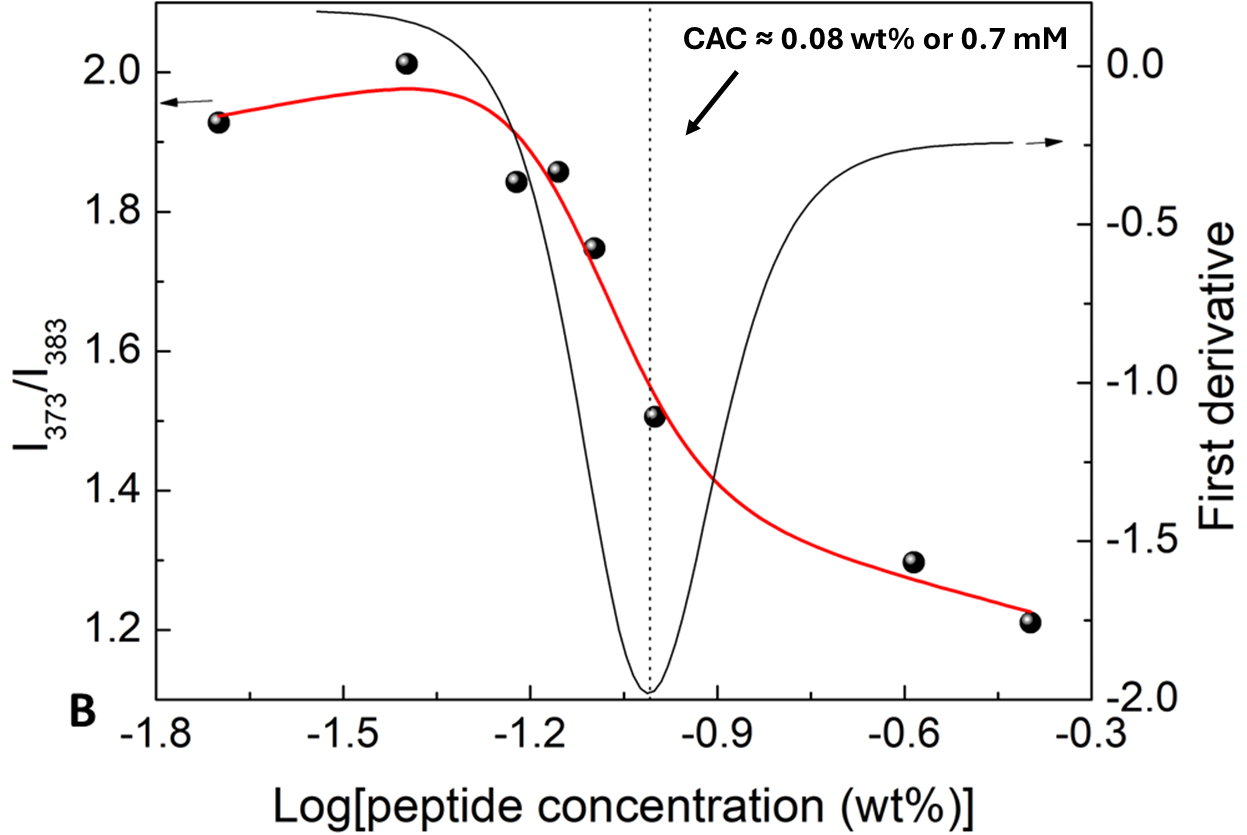
**

**Figure S2**: Fluorescence assays for different concentrations of peptide in a solution of 50 uM of pyrene, the fluorescence intensity ratio being fitted using a derivative function, which places the CAC around 0.08 wt%, equivalent to 0.7 mM (0.8 mg/mL).


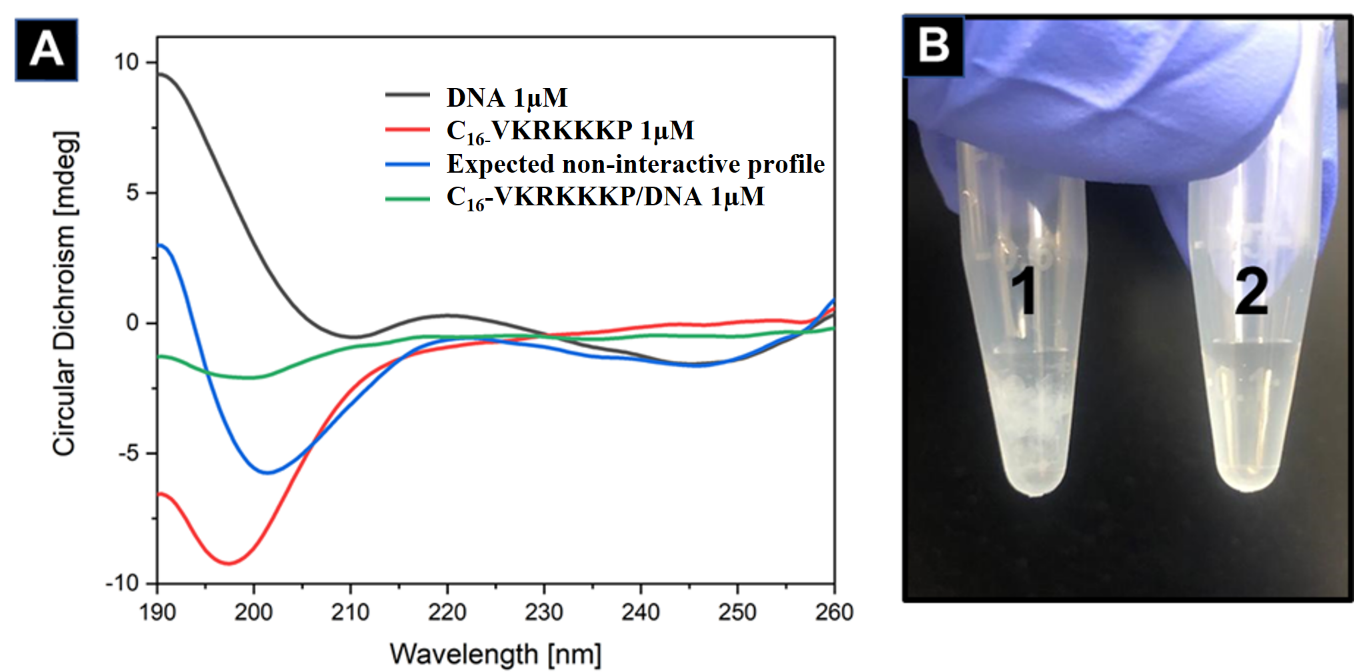


**Figure S3:** (A) CD spectra of DNA 1 mM (black curve), C_16_-VKRKKKP at 1 mM (red curve). To identify a possible structuration between the molecules, a spectrum was obtained from the addition of the CD spectra of DNA and C_16_-VKRKKKP at 1 mM curves as a simple simulation of a non-interacting mixture (blue curve) compared to the measured CD spectrum of a solution containing C_16_-VKRKKKP/DNA at 1 mM (green curve). (B) Images of samples. Tube **1** contains a solution of C_16_-VKRKKKP/DNA at 1 mM, and tube **2** contains only the peptide amphiphile. It is possible to observe a difference in turbidity between the two samples, possibly due to phase separation and precipitation in the first tube.

**Figure S4:** 1D plot from a fibre X-ray diffraction pattern obtained from a sample of C_16_-VKRKKKP/DNA at a ratio of 2:1. The data shows a prominent peak at 4.2 Å. The initial samples used for fiber production were at a concentration of 10 mg/mL of C_16_-VKRKKKP.

Flow cytometry was used to quantify the internalization of the fragmented DNA/YOYO-1 labelled. The gate strategy adopted is shown below.


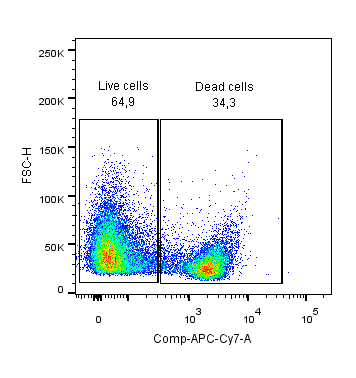

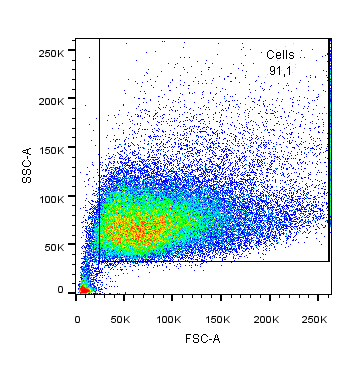


FSC-A

SSC-A


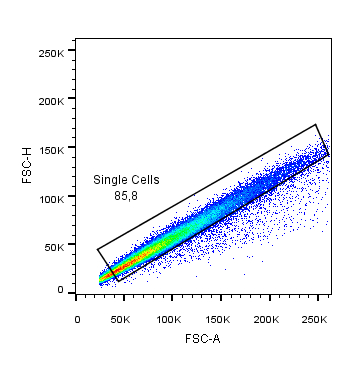


FSC-H

FSC-A

Fixable Dye


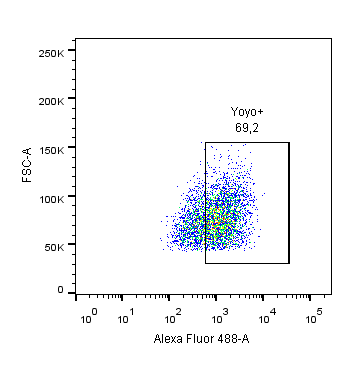


FSC-A

Alexa Fluor 488

FSC-A

FSC-H

**Figure S5: Gate strategy used to evaluate the transfection rate (YOYO-1^+^ cells).** The HeLa cells were selected by complexity (SSC-A) and size (FSC-A). The doublets and cell aggregates were excluded using the combination of FSC-H and FSC-A. The dead cells were identified and excluded using a live/dead dye labelled with APC-Cy7. Finally, the Yoyo-1^+^ cell population were identified as Alexa Fluor 488^+^ by the software FlowJo X, which presents a similar λex/λem. to YOYO-1

Since the samples were incubated in plates of 24 wells, enabling the seeding of 5×10^4^ cells per well, we defined a minimum cut-off of 5×10^3^ events per sample (at least 10% of the total sample, for a representative analysis). The experiments were repeated independently at least one more time, with similar results as shown in the last panel of Figure S6.


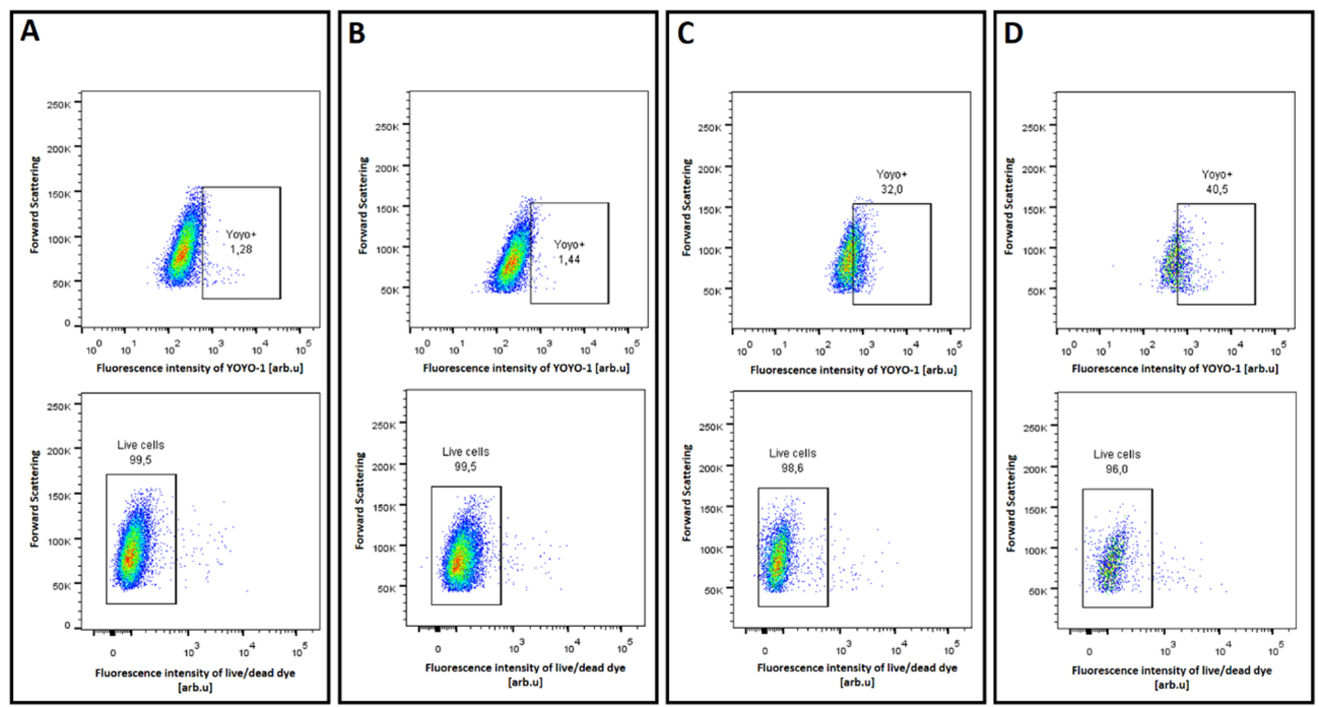


**Figure S6**: Dot plots of flow cytometry assays delimiting gates for populations of HeLa cells positive for YOYO-1 fluorescence (first row) and dead cells positive for the fixable viability dye (second row). The cells were incubated only with DNA (A), peptiplexes at a 2:1 ratio of VKRKKKP /DNA (B), 1:1 C_16_-VKRKKKP/DNA (C) and 2:1 C_16_-VKRKKKP /DNA (D). We also observed a difference in the translocation of peptiplexes (dyed in green by the fluorophore YOYO-1 used to label the fragmented DNA, blue staining of nuclei with DAPI) by confocal microscopy, with a lower degree of internalization by the original VKRKKKP and a higher delivery for C_16_-VKRKKKP.

Although higher ratios of peptide/DNA presented higher values of (3:1, 5:1 and 10:1) compared with 2:1 and 1:1 mixtures, it also led to larger morphological changes and aggregation for those cells, failing to achieve or cut-off of 5×10^3^ events observed per sample, at least twice. An example of this can be found in Figure S7A, where a decrease in the number of events due to cell aggregation and morphogenesis can be observed. This change in cell morphology with a decrease in size and rounder cells can be observed in Figure S7


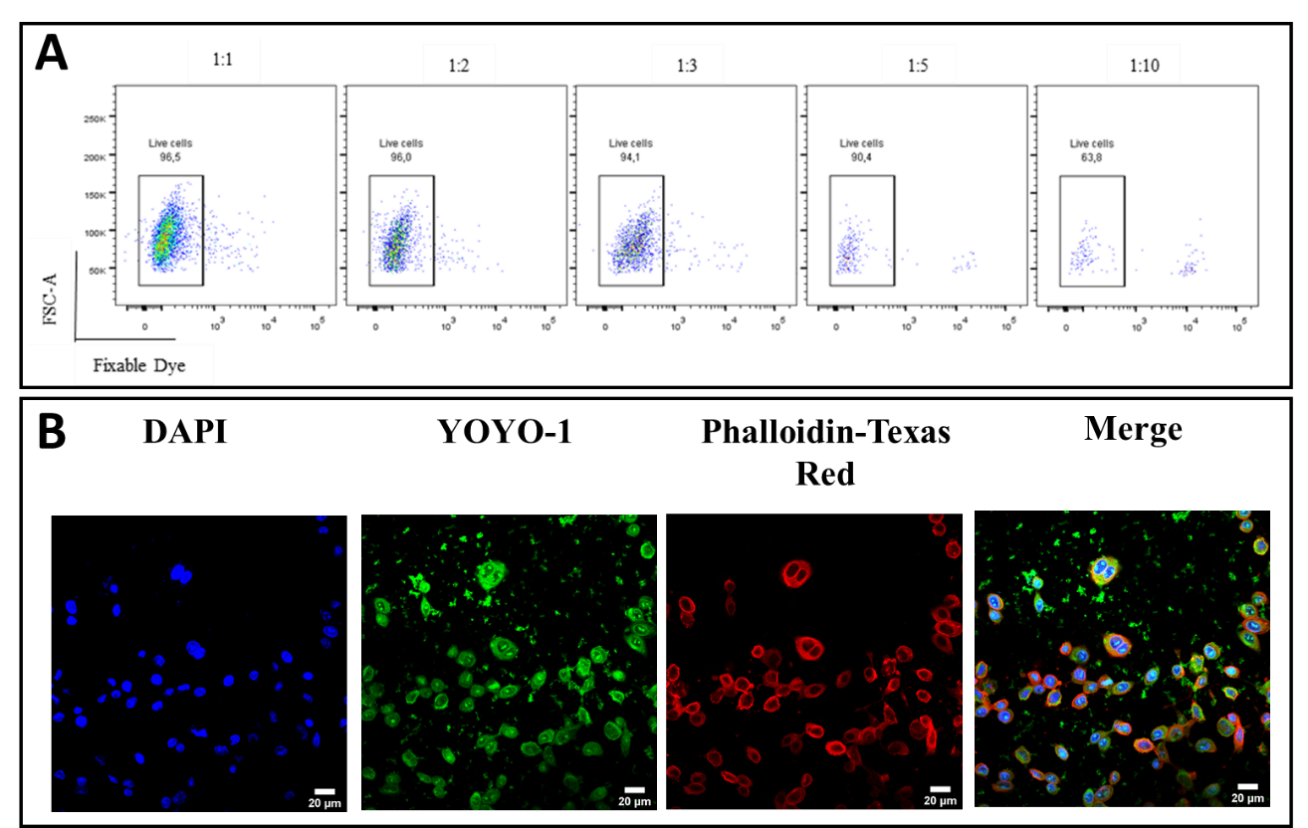


**Figure S7:** A) Representative dot plots of viability analysis of cells transfected with different ratios of C_16_-VKRKKKP. In flow cytometry analysis it was observed that at higher ratios of peptide/DNA (3:1, 5:1 and 10:1), cells underwent further morphogenesis and aggregation, which can be seen in B) in which HeLa cells presented a rounder morphology with a decrease in size and a tendency to aggregation. Those events were excluded from the analysis, resulting in a few events (n) inferior to our cut-off of 5×10^3^.


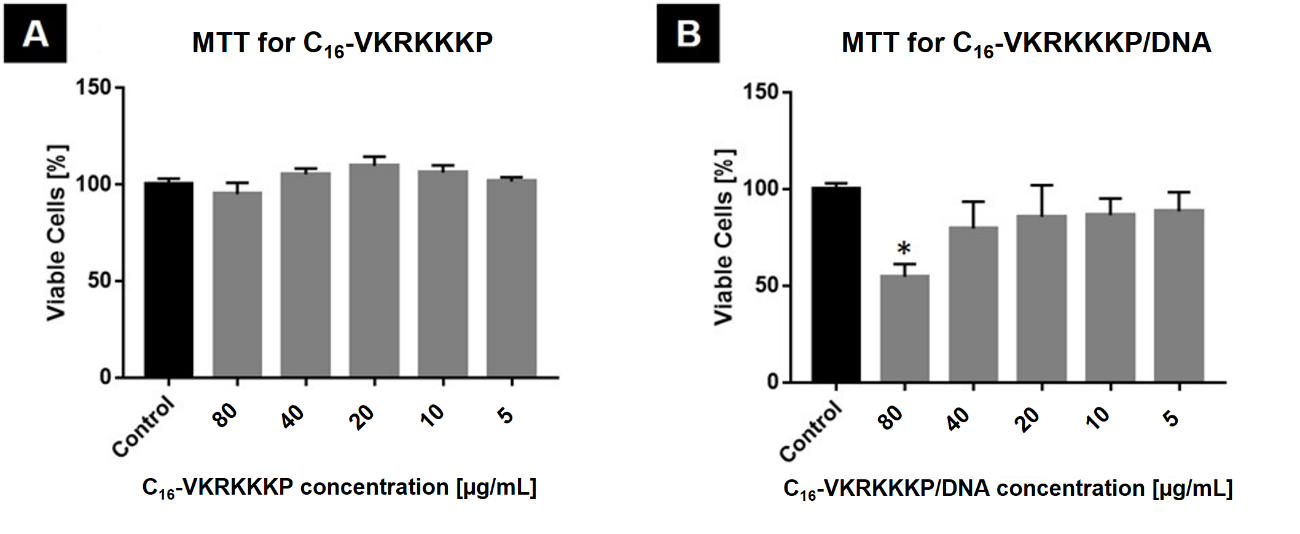


**Figure S8**: (A) MTT without DNA. (B) MTT with peptiplexes between C_16_-VKRKKKP and fragmented DNA. The peptiplexes were slightly more toxic when compared with samples incubated without DNA. There was a significant difference in cell viability between the control the data for 80 μg/mL, as indicated by a Kruskal-Wallis analysis, n =3 and p < 0.05.

**
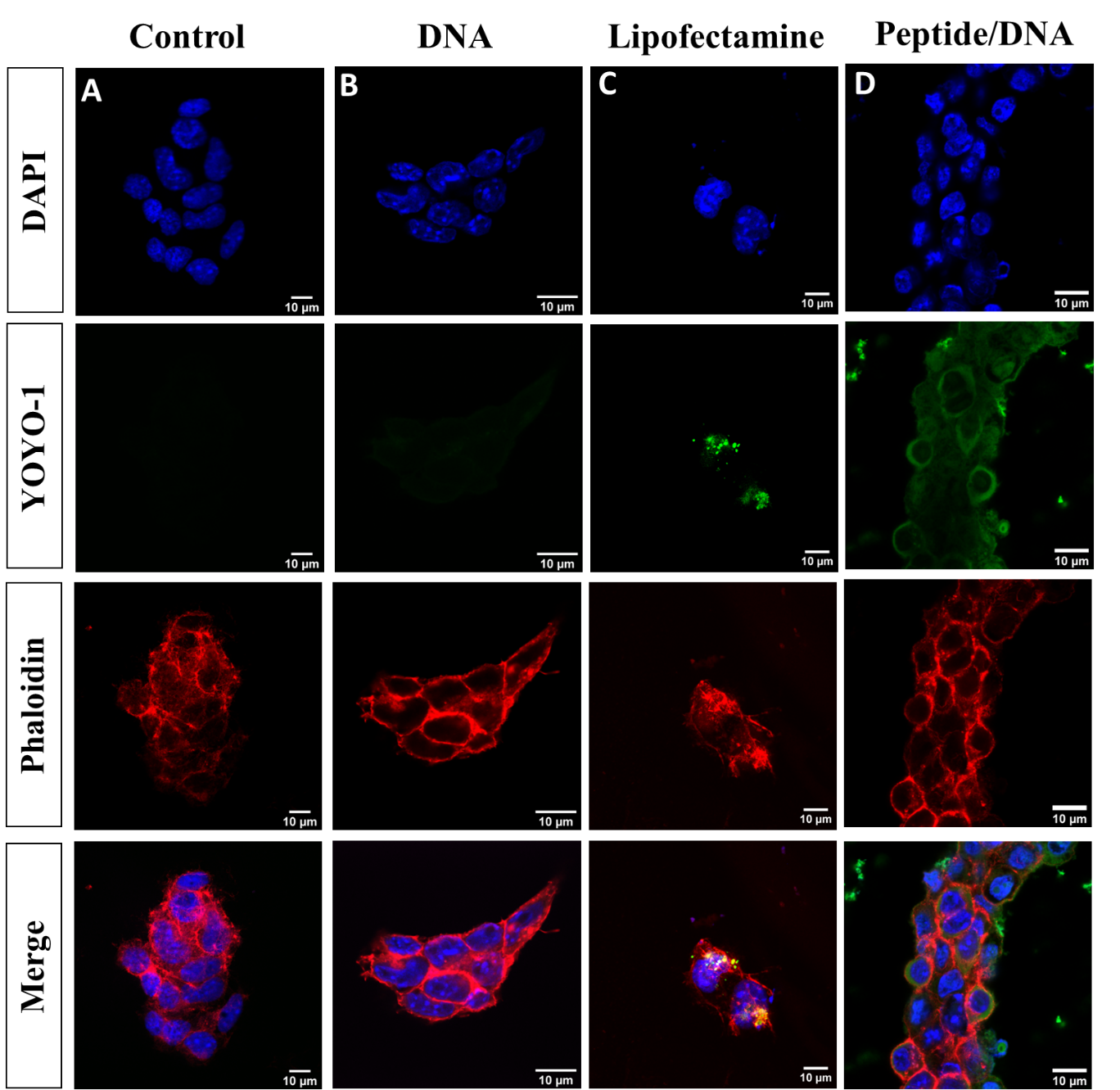
**

**Figure S9:** Confocal images from ES-E14TG2a cells incubated for 4 hours with (A) only DMEM without serum (B) 5 µg of fragmented DNA labelled with YOYO-1 (C) Lipofectamine 2000 + 5 µg of labelled DNA and in (D), peptiplexes of C_16_-VKRKKKP /DNA at 2:1 ratio. The samples incubated only with DNA did not present any significant fluorescence of YOYO-1, contrasting with the strong green fluorescence of samples incubated with DNA and lipofectamine or of C_16_-VKRKKKP.

**Table S1.** Best fit parameters for SAXS data. A flat background was used in all models. Electron densities were arbitrarily set to -1 and 1, respectively, for cores and shells with an overall intensity scaling factor. R = cylinder radius; R_i_ = inner radius of the spherical shell; R_o_= outer radius of the shell; R_g_= gyration radius of the chain; ν = Flory exponent; σ_H_= width (standard deviation) of the lipopeptide polar head; σ_c_ = width of the bilayer core; *t* = head-to-head separation in bilayer

| **Model** | **Porod cylinder^i^** | **Spherical shell^ii^** | | **Gaussian chains^iii^** | | **Gaussian bilayer^iv^** | | |
| --- | --- | --- | --- | --- | --- | --- | --- | --- |
| Parameter | R  (nm) | R_i_  (nm) | R_o_  (nm) | R_g_  (nm) | ν | σ_H_  (nm) | σ_c_  (nm) | t  (nm) |
| **1 mg/mL DNA** | 1.1 ±0.3 |  |  |  |  |  |  |  |
| **5 mg/mL PA** |  | 1.8 | 2.1 |  |  |  |  |  |
| **10 mg/mL PA** |  |  |  | 0.8 | 0.33 | 1.7 | 1.6 | 5.5 |
| **0.5 mg/mL PA + 0.1 mg/mL DNA*** | 3.9 | 2.1 | 3.5 |  |  |  |  |  |

*For the lipopeptide/DNA data, a power law was introduced to describe the Porod interface at low q (exponent = -4) and the length of the cylinder (L) was fixed at L = 100 nm.

Models applied from sasfit:^1-2^ Porod Cylinder^i^, Spherical Shell I^ii^, Generalized Gaussian Coil^iii^ and BiLayerGauss^iv^

**References**

(1) Bressler, I.; Kohlbrecher, J.; Thünemann, A. F., SASfit: a tool for small-angle scattering data analysis using a library of analytical expressions. *J. Appl. Cryst.* **2015,** *48*, 1587-1598.

(2) Kohlbrecher, J.; Bressler, I., Updates in SASfit for fitting analytical expressions and numerical models to small-angle scattering patterns. *J. Appl. Cryst.* **2022,** *55*, 1677-1688.
